# Ecological Network Inference From Long-Term Presence-Absence Data

**DOI:** 10.1038/s41598-017-07009-x

**Published:** 2017-08-02

**Authors:** Elizabeth L. Sander, J. Timothy Wootton, Stefano Allesina

**Affiliations:** 1 University of Chicago, Department of Ecology and Evolution, Chicago, 60637 USA; 2 University of Chicago, Computation Institute, Chicago, 60637 USA

## Abstract

Ecological communities are characterized by complex networks of trophic and nontrophic interactions, which shape the dy-namics of the community. Machine learning and correlational methods are increasingly popular for inferring networks from co-occurrence and time series data, particularly in microbial systems. In this study, we test the suitability of these methods for inferring ecological interactions by constructing networks using Dynamic Bayesian Networks, Lasso regression, and Pear-son’s correlation coefficient, then comparing the model networks to empirical trophic and nontrophic webs in two ecological systems. We find that although each model significantly replicates the structure of at least one empirical network, no model significantly predicts network structure in both systems, and no model is clearly superior to the others. We also find that networks inferred for the Tatoosh intertidal match the nontrophic network much more closely than the trophic one, possibly due to the challenges of identifying trophic interactions from presence-absence data. Our findings suggest that although these methods hold some promise for ecological network inference, presence-absence data does not provide enough signal for models to consistently identify interactions, and networks inferred from these data should be interpreted with caution.

## Introduction

Species interactions are a major driver of population dynamics. Interactions may produce predator-prey^1^ or host-pathogen^2^ cycling, competitive exclusion^3^, or complex dynamics resulting from multiple interactions^4, 5^. In general, it is easier to collect population abundance data than observing interactions directly; as a result, a major goal in ecological modeling is the inference and study of ecological interactions based on spatio-temporal population dynamics of species. Mechanistic models are a popular approach, and can provide a way to reveal underlying biological processes^6^. However, these models often require detailed information about the system, including estimates of underlying parameters, or otherwise require computationally intensive stochastic simulations to infer these parameters^7^. To study the interactions between more than a few species, it is necessary to find a more scalable approach.

At the community level, ecological interactions produce a complex network. Trophic web (food web) and interaction web structures affect dynamical properties such as community stability^8, 9^, reactivity^10^, and robustness to extinction^11, 12^. As with individual two-species interactions, we expect the structure of interaction networks to be reflected in the patterns of community abundance through time. Whole network inference from community population dynamics is challenging using standard mechanistic approaches. State-space reconstruction has been suggested as a model-free method for inferring pairwise ecological interactions^13, 14^, but has been critiqued for its sensitivity to common features of natural systems, such as process error^15^.

Machine learning and correlational methods have increasingly been used to infer microbial^16, 17^ and gene expression^18–20^ networks from time-series and co-occurrence data, and have been proposed as an approach to biomonitoring^21^. These methods stand in contrast to the classical approach of observing or counting interactions commonly used in macroscopic communities (*e.g*. refs 22 and 23). Machine learning and correlational methods are convenient in that underlying parameters need not be estimated or even specified; instead, the model is fit based on observational data alone, and, when possible, tested using cross-validation or out-of-sample data (data that is used not to fit the model, but to test its predictive accuracy). This process produces “association networks” that reflect co-occurrence or correlational patterns. The hope is that these association networks reflect ecological dynamics, based on the much-debated theory that nonrandom co-occurrence distributions result from ecological interactions^24–26^. Methods such as joint and latent variable modelling have similar aims, but with more of a focus on predicting abundance and studying correlation structure than inferring a binary network of interactions^27^.

Experimentally or observationally verified microbial interaction networks are not commonly available, so it is difficult to determine if these methods effectively capture ecological interactions^16^. Macroscopic communities are useful to study here, because a combination of observational, experimental, and network data are available for a few well-studied systems. Testing the validity of inferred networks for these systems can benefit both microbial ecology and traditional community ecology. If the models produce high-quality networks, we can have more trust in these methods in systems at either scale; if the networks do not correspond to empirical knowledge, we need to be cautious in interpreting the association networks currently being produced for microbial systems. Only one study to our knowledge has applied network inference methods to macroscopic ecological systems^28^. This study found that Bayesian networks successfully identified many known interactions among species and habitat variables. We expand on this work by considering presence-absence data for multiple large communities, using time series data for dynamic rather than static methods.

It is important to consider what the inferred co-occurrence networks mean ecologically. An inferred link represents a predictive or correlational link between one species and another species in the future, links which do not necessarily require interaction or causality. However, it seems reasonable to expect that when two species interact, that interaction will be reflected in the population dynamics, and therefore, that we could identify many interactions based on observed population dynamics. Presence-absence data dampens this dynamical signal, making it more challenging to correctly infer interactions. With this weaker signal, it may be easier to infer some types of interactions better than others. For example, predator-prey interactions tend not to cause total extinction of either species, and therefore might be difficult to identify from presence-absence alone. In contrast, competition can result in local extinction from competitive exclusion, and facilitation may allow for the successful invasion of a locally absent species. For this reason, we expect nontrophic interactions such as competition and facilitation to be more easily identified than trophic ones.

We use three network inference methods, which represent major classes of algorithms used to construct gene and microbial networks. The first approach uses Dynamic Bayesian Networks (DBNs), which uses patterns of conditional independence between variables to infer a graphical structure. The second is Lasso regression, which constrains the inclusion of regression coefficients in the model. The final method uses Pearson’s correlation coefficients with a significance threshold. All methods are presented in detail in the methods. Correlational approaches are especially common in microbial ecology^29–32^, and are distinct in that they are not predictive models (*i.e*., the Pearson’s correlation coeffient method can be used to construct an association network, but not to predict future population dynamics, as DBNs and Lasso can). We train the models to predict which species are present based on which were present in the previous sample time. This allows us to capture the relationships between species through time, rather than assuming that species respond to each other instantaneously.

To evaluate how successfully these three models infer binary networks from ecological data, we apply them to two long-term presence-absence datasets: a riverine fish community in France, and a diverse intertidal community on Tatoosh Island in Washington state. These datasets differ in several ways: the France dataset spans a larger spatial scale, but contains fewer species and less phylogenetic diversity than the Tatoosh system. Since it includes only fish, it might be considered a guild, rather than a complete community. This allows us to evaluate the robustness of these inference methods under different data scenarios, and for different types of communities. We consider how well the models infer both trophic networks in both systems, and the nontrophic network from the Tatoosh intertidal. We find that each class of model inference infers at least one network structure which is more similar to the empirical network than random, but that no class of model does so for both France and Tatoosh, and no method outperforms the others in general. We also find that the models are completely unable to capture the Tatoosh trophic network, but that multiple models infer networks which are similar to the Tatoosh nontrophic network, suggesting either that nontrophic interactions drive population dynamics in this system, or that the dynamical signal of trophic interactions gets lost when using presence-absence data. Overall, although these methods have some predictive ability even with the limitation of presence-absence data, they should be used and interpreted with care.

## Methods

We used multi-site, presence-absence, time series datasets from two systems: stream fish in France, and the middle intertidal zone on Tatoosh Island. We predicted binary network structures using three approaches: Dynamic Bayesian Networks, Lasso regression, and a method based on Pearson’s correlation coefficients. We then compared the model-inferred network structures to networks based on empirical data. For the two predictive models (DBNs and Lasso), we also used the models to make short-term (next time step) predictions using out-of-sample data; that is, data which were not used to fit the model, and were set aside to test prediction accuracy. Code used to analyze these data is available on GitHub at https://git.io/vDEFu.

### Data

The French stream fish dataset is a long-term monitoring dataset collected by ONEMA, the French National Agency for Water and Aquatic Environments. We used the data available on Dryad^33^, which includes presence-absence information for 32 common species of fish, collected at 794 sites from 1992 to 2011, and split into eight time periods, with each time period spanning either two or three years. Not all sites contained data from all time periods. Because we used models that relied on the time series nature of the data, only consecutive sets of time points were used. For more information on how these data were collected, see refs 34 and 35.

We constructed a trophic network for these species based on gut content analyses from 88 articles. This count excludes studies that only identified cannibalistic interactions, only identified interactions with species not present in the network, or did not identify prey to the species level. Feeding interactions were incorporated into the network if they were observed in at least one study. Prey items were excluded when identity was unclear or not resolved to the species level, and when interactions were based on a single or spurious observation. Consumption of eggs, larvae, and fry were included as interactions if they were identified to the species level. Cannibalistic interactions were excluded from the trophic network, since self-interactions in the inferred networks mostly represent autocorrelation rather than cannibalism. Since this dataset contains only fish, all interactions in this network represent piscivory. While it is possible that this literature-based trophic network is missing some interactions, all species in the dataset are quite common and many articles containing dietary information were available. Another possible concern is that the “sampling effort” is different for different species, since species may differ in interest for research or fishery purposes. Fortunately, many gut content analyses were available for most fish, especially for highly piscivorous species. Of all species in the web identified as partially or entirely piscivorous on FishBase^36^, we only had difficulty finding dietary information for the barbel *Barbus barbus*, which we consequently removed from both the network and time-series data. A listing of the feeding interactions and the citations supporting them can be found in Table S3 and on Dryad^37^.

The Tatoosh Island presence-absence dataset is a long-term observational and experimental dataset from the middle intertidal zone, non-destructively sampled at 30 plots annually. This zone is extremely diverse, and the dataset includes sessile species such as mussels, barnacles, and algae, along with mobile species such as echinoderms, limpets, and snails. For a full list of species, see the supporting data on Dryad. Ten control and ten experimental removal plots were sampled from 1994 to 2012, with an additional five experimental plots sampled from 1998 to 2012. In the experimental plots, the competitive dominant *Mytilus californianus* was chronically and selectively removed. Censuses were performed in each plot by recording percent cover (sessile species) or numbers seen (mobile species) with the aid of a quadrat subdivided with microfilament lines into 121 sub-squares. For further details on the census methods, see refs 38 and 39. Census data were converted to presence-absence after collection.

The middle intertidal zone is dominated in biomass by the mussel *Mytilus californianus*, which grows in dense mats. These mats are occasionally torn from the rock by strong wave action, revealing patches of bare rock, which then follows a predictable successional pattern of colonizing species, before being taken over by *M. californianus* again^40, 41^. The mussel beds provide physical structure for a diverse set of sessile and mobile species. The non-destructive nature of the sampling procedure makes it possible to study the undisturbed dynamics of the community, but as a result may miss some species that live deeper in the mussel bed. 107 species were identified across 530 site-years, but only the 60 species which were observed 10 or more times were used in the analysis.

The composition of plots with and without mussels is strikingly different, with control plots generally dominated by *M. californianus*. *Mytilus californianus* is so dominant in these plots that it may mask dynamics between other species. There is no single dominant species across the experimental plots, and including these data may reveal species interactions that would be hard for a model to identify from the control data alone. We attempted to fit separate models for the control and experimental plots, but the data were too limited. Because *M. californianus* removal has been found to dampen environmental stochasticity, but not to affect the temporal order of the dynamics^38^ (that is, it changes the size of fluctuations in the system, but not the timescale over which they occur), we believe that it is reasonable to model the system using both experimental and control plots.

Interaction data for Tatoosh Island were collected based on observational and natural history information obtained in nearly a half century of study at the site^42^. We used two versions of the network: a trophic web, with only feeding interactions, and a nontrophic web with only non-feeding interactions, including competition, mutualism, facilitation, commensalism, and amensalism.

### Model Training and Cross-Validation

Networks were inferred using three methods: Dynamic Bayesian Networks, Lasso regression, and Pearson’s correlation coefficient. Models were fit using training data, and tested for predictive ability on a separate set of test data. Since the method based on Pearson’s correlation coefficients does not produce a predictive model, it was used only to fit a network structure. With the exception of DBN structure learning, all model fitting and prediction was performed in R^43^ using the package glmnet for Lasso regression^44^ and helper functions from several other packages^45–53^.

The France dataset was split into training and test sets (60 and 40 percent of sites, respectively). The Tatoosh dataset was small enough (30 sites with annual samples for 15 or 19 years each) that setting aside a test set was not feasible. Instead, one data point was randomly excluded from each site. The model was fit to the remaining data, and the excluded points were used as a small test set. This procedure was performed 14 times, such that all data points but the first year served as an out-of-sample point at most once. The first year could not be used for out-of sample prediction since the network models use data from the previous time point to make predictions. Following this procedure, 14 separate models were fit for each method, so that a distribution of model performance could be obtained.

### Static and Dynamic Bayesian Networks

A Bayesian Network is a probabilistic model that uses a graphical structure to represent relationships between the random variables. The structure ${\mathscr{G}}$ contains vertices ${\mathscr{V}}$ and directed edges ${\mathcal E}$. The vertices (nodes) represent the random variables whose relationships are under consideration, in this study, presence/absence of species. Edges (links) represent conditional relationships from one node (the “parent”) to another (the “child”); put more precisely, the absence of an edge between two nodes represents a *conditional independency* between the two nodes. The probability distribution of a child node *C* given parents ***P*** is conditionally independent of all other vertices in ${\mathscr{G}}$; that is, $P(C|{\mathscr{V}})=P(C|{\boldsymbol{P}})$.

These conditional independencies allow the joint probability distribution of ${\mathscr{G}}$ to be given in terms of simple conditional probabilities. The joint probability distribution of ${\mathscr{G}}$ for nodes *X*_1_, *X*_2_, …, *X*_*n*_, with parents ***P***_*i*_, is given by

1 $$P({X}_{1},{X}_{2},\ldots ,{X}_{n})=\prod _{i\mathrm{=1}}^{n}P({X}_{i}|{{\boldsymbol{P}}}_{i})$$

Bayesian networks of this kind, which do not have a time component, are known as *Static Bayesian Networks* (SBNs). SBNs are limited by a statistical constraint: the network must be a directed acyclic graph (DAG); that is, it may not contain directed cycles. This means that if species A depends on species B, B may not depend on A. This does not conform to ecological reality at all. For example, since predators gain biomass by consuming prey, and prey populations are reduced by predation, we expect predators and prey to mutually influence each other, as is reflected in dynamical ecological models such as Lotka-Volterra^1^.

**Figure 1:**
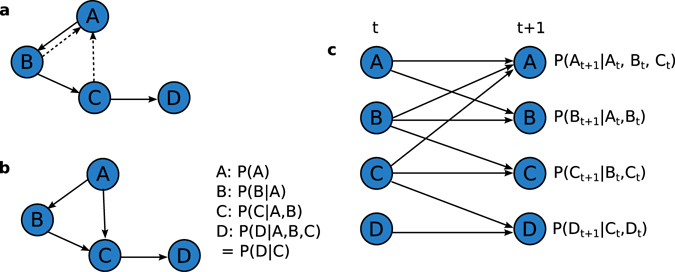
Example of Static and Dynamic Bayesian Network structures. (**a**) Shows a “true” network of dependencies between four example nodes, which contains two directed cycles: $A\to B\to A$ and $A\to B\to C\to A$. Since this is not a DAG, we cannot capture the true structure of this network with an SBN. Removal of the dashed edges would create a valid DAG. (**b**) Shows a valid DAG that could be used as an SBN structure for these nodes, created by removing the $B\to A$ link and flipping the direction of the $C\to A$ link. The local probability distributions that define the SBN are shown to the right. (**c**) Shows the true network “unfolded” in time into a DBN structure. This unfolded structure captures all dependencies between nodes in the true network, but is a valid DAG. Nodes at time $t$ are on the left, nodes at time *t* + 1 on the right. Self-dependencies have been included to capture autocorrelation. Local probability distributions that define the DBN are given to the right of each node.

We can create more ecologically reasonable networks by “unfolding” the network in time, creating a *Dynamic Bayesian Network* (DBN)^54^. Using this framework, the presence/absence of a child species at time *t* + 1 depends on the presence or absence of its parent species at time *t*. Put more precisely, the probability that the child species is present at time *t* + 1 is conditionally independent of all non-parent species, given the presence/absence of the parent species at the previous time step *t*. Since parents always predict the child in the next time step, any species may be a parent for any other without violating the DAG constraint. A species may also be its own parent, capturing the effects of autocorrelation. For a DBN, the joint probability of ${\mathscr{G}}$ may be given as follows:

2 $$P({X}_{\mathrm{1,}t+1},{X}_{\mathrm{2,}t+1},\ldots ,{X}_{n,t+1})=\prod _{i\mathrm{=1}}^{n}P({X}_{i,t+1}|{{\boldsymbol{P}}}_{i,t})$$

where ${X}_{i,t+1}$ is node *X*_*i*_ at time *t* + 1, and ***P***_*i,t*_ is the set of parents ***P***_*i*_ at time *t*. For notational simplicity, we drop the subscript for time in future equations. The state of the parents is always given for time *t*, and the child or random variable is always given for time *t* + 1. For an example of how an empirical network may be represented by Static and Dynamic Bayesian Networks, see Fig. 1.

#### Structure Learning

The model as described above assumes that the structure ${\mathscr{G}}$ is known. Since we are trying to infer a network structure from our data, we also need to learn the structure of ${\mathscr{G}}$. In theory, this can be done in a fully Bayesian way, by calculating a posterior distribution over both the parameter and network structure space^55^. In practice, this approach is intractable, and it is necessary to use a search algorithm to identify high-quality network structures (which are not guaranteed to be optimal), and evaluate them using a score proportional to the posterior probability of the structure given the data. We use the commonly-used scoring metric Bayesian Dirichlet equivalence (BDe)^56^. We used the software Banjo^57^ for structure learning, using simulated annealing as a search algorithm, with 100 runs for 8 hours each, for each dataset. For the Tatoosh dataset, this includes 100 8-hour runs for each of the 14 subsampled datasets. The best network from each run was saved.

Due to the computational complexity of the problem, structure learning in Banjo requires that a maximum number of parents per child node be set. As a check on the sensitivity of the results to this constraint, the structure learning process and all subsequent analyses were repeated for 2, 3, 4, and 5 maximum parents.

Consensus networks were built from the saved networks. For the France dataset, a majority rule consensus network was built using the top 10 networks. The consensus network was used both for predicting out-of-sample data and for comparison to the empirical network structure. The process was slightly different for the Tatoosh dataset, because of the multiple subsampled datasets. For each subsampled dataset, a majority rule consensus network was built using the top 10 networks. These consensus networks were used to predict out-of-sample data points. To create a single network for comparison to the empirical network structure, a majority rule consensus network was built from the 14 subsampled consensus networks, resulting in a “consensus-consensus” network. Search algorithm convergence was examined using the percentage of links that differed between the consensus networks and each network used to compute the consensus. The percentage difference *d* between two networks *A* and *B* was calculated as

3 $$d=\frac{{(A/B)}^{L}+{(B/A)}^{L}}{{A}^{L}+{B}^{L}}$$

where *A* ^*L*^ is the number of links in network *A*, *A/B* is the relative complement of *B* with respect to *A* (the links in *A* but not *B*), and (*A/B*)^*L*^ is the number of links in *A/B*. For the France dataset, *d* was calculated between each consensus network and the top 10 networks used to create it. Similarly, for the Tatoosh dataset, *d* was calculated between each consensus network and the top 10 networks used to create it, for each of the 14 data subsamples.The distribution of *d* provides a sense of how similar top solutions are, and by extension, how well the search algorithm is converging on optimal or close-to-optimal solutions.

#### 0.0.1 Posterior Calculation and Prediction

After inferring a structure ${\mathscr{G}}$ as described above, we calculated the joint posterior distribution of the DBN model given ${\mathscr{G}}$. To calculate the joint posterior distribution, we can use the local distribution functions $P({X}_{i}|{{\boldsymbol{P}}}_{i},{\mathscr{G}})$ for each Bernoulli random variable *X*_*i*_. Because each parent may take on one of two states (0, “absent”, or 1, “present”), there are ${2}^{{k}_{i}}$ possible configurations of the parents, where *k*_i_ is the number of parents for species *i*. Thus, each local distribution is a collection of binomial distributions, one for each parent configuration *j* (that is, ${{\boldsymbol{P}}}_{i}=\{{{\boldsymbol{p}}}_{ij}\}$ ^58^).

We can analytically calculate a posterior distribution given these local distribution functions with two additional assumptions. The first is that the data in our random sample *D* is complete (that is, no data are missing), which is true in both datasets we considered. The second is that all parameters *θ*_*ij*_ are mutually independent, where *θ*_*ij*_ is the probability species *i* is present in a Bernoulli trial given parent configuration ***p***_*ij*_. The assumption of parameter independence is demonstrated for multinomial local distribution functions in Heckerman 2008^58^, which also applies to the more restricted case of binomial local distribution functions.

For any node *i* and parent state ***p***_*ij*_, the likelihood of the observed data is given by the likelihood function for binomial sampling:

4 $$ {\mathcal L} ({\theta }_{ij})\propto {\theta }_{ij}^{{r}_{ij}}{\mathrm{(1}-{\theta }_{ij})}^{{n}_{ij}-{r}_{ij}}$$

where *r*_*ij*_ is the number of times *X*_*i*_ was present in the data given state ***p***_*ij*_ in the previous time step, and *n*_*ij*_ is the total number of data points with the parent state ***p***_*ij*_ in the previous time step.

To make this analytically tractable, we can choose a Beta prior on the parameter *θ*_*ij*_, such that

5 $$P({\theta }_{ij}|{\mathscr{G}})=\frac{{\rm{\Gamma }}({\alpha }_{ij}+{\beta }_{ij})}{{\rm{\Gamma }}({\alpha }_{ij}){\rm{\Gamma }}({\beta }_{ij})}{\theta }_{ij}^{{\alpha }_{ij}-1}{\mathrm{(1}-{\theta }_{ij})}^{{\beta }_{ij}-1}$$

where ${\alpha }_{ij}$ and ${\beta }_{ij}$ are hyperparameters which determine the shape of the prior distribution. We choose ${\alpha }_{ij}\mathrm{=1}$ and ${\beta }_{ij}\mathrm{=1}$ for all nodes *i* and configurations *j*. This produces a flat prior, which corresponds to a level of confidence equivalent to observing *X*_*i*_ to be present once and absent once. Since the Beta distribution is a conjugate prior to the binomial likelihood function, the posterior distributions are simply given by

6 $$P({\theta }_{ij}|D,{{\boldsymbol{p}}}_{ij},{\mathscr{G}}) \sim Beta({\theta }_{ij}|1+{r}_{ij}\mathrm{,1}+{n}_{ij}-{r}_{ij})$$

Having calculated the posterior distributions, we can then use them to make predictions about the state of the system at time *t* + 1, given the state at time *t*. Using data from time step *t* to establish the state of the parents, the expected probability that species *i* is present at time *t* + 1 is given by the expectation of the Beta distribution for the parent state ${{\boldsymbol{p}}}_{ij}$:

7 $$P({X}_{i}=1|D,{{\boldsymbol{p}}}_{ij},{\mathscr{G}})=\frac{1+{r}_{ij}}{2+{n}_{ij}}$$

Repeating this process for all species, we can predict the state of all species in the next time step. In this way, we can compare the predicted state to the observed state at each timepoint in the out-of-sample (test) data, in order to evaluate the short-term predictive power of the model.

### Lasso Regression

Lasso regression is a generalized linear regression approach with an additional regularization parameter *λ* ^59^. This regularization parameter acts to shrink or eliminate predictor coefficients that contribute least to the model fit, helping to prevent overfitting. The line of best fit is calculated based on the following equation:

8 $$(\hat{\alpha },\hat{{\boldsymbol{\beta }}})=argmin\{\sum _{i\mathrm{=1}}^{N}{({y}_{i}-\alpha -\sum _{j}{\beta }_{j}{x}_{ij})}^{2}\}\,{\rm{where}}\,\sum _{j}|{\beta }_{j}|\le \lambda $$

where $\hat{\alpha }$ is the intercept for the line of best fit, $\hat{{\boldsymbol{\beta }}}$ is the vector of coefficients for the line of best fit, *N* is the number of data points in the random sample, ${y}_{i}$ is the observed value of the dependent variable for the *i*th data point, and *x*_*ij*_ is the observed value of the *j*th predictor for the *i*th data point. As in a standard generalized linear model, we are minimizing the sum of squared errors, but with an additional constraint on the total magnitude of the *β*s. This causes the *β*s for relatively unimportant predictors to shrink to 0. We used this shrinkage property to construct a network. By running Lasso regression on each species, we identified the interacting species with the strongest predictive effect, and created a network from those interactions. Lasso was chosen over other regularization approaches (such as elastic net) due to its popularity for biological network inference^17, 20, 28, 60^.

A separate regression was performed for each species, where the presence/absence of one species was regressed on the presence/absence of all species (including itself) at the previous time step; that is, the lag 1 of all species. Predictor species with non-zero coefficients were kept as interactions in the resulting network. We used *k*-fold cross-validation with the training set to identify the optimal value of *λ*. Using this value, we used the trained model to predict the test set. We trained two Lasso regression models: one containing only first-order (*i.e*., pairwise) interactions, and one containing both first and second-order interactions. For the model containing second-order interactions, any pair of species included in a second-order interaction were considered to be interacting, for purposes of constructing the network.

The DBN model is a Bayesian approach, and therefore does not have a specific amount of data required to train a model. However, logistic regression models, similar to the lasso regression used here, can produce unstable results when there are too few observations in each category^61^. To avoid this issue, we excluded species with fewer than 20 observations in each class from the analysis (species which were chronically present or chronically absent). No species from the France dataset were excluded, but 8 species were excluded from the Tatoosh dataset for this reason: *Semibalanus cariosus*, *Leathesia*, *Prionitis*, *Lottia pelta*, *Lottia digitalis*, *Tonicella lineata*, *Pinnotheres pisum*, and *Henricia*. These species were also excluded from the Tatoosh empirical networks when comparing them to the inferred Lasso networks.

### Pearson’s Correlation Coefficient Method

We calculated the Pearson’s correlation coeffient for each species with the presence/absence of each other species in the previous time step (that is, we calculated the correlation between each species and the lag 1 of each other species). We then performed a permutation test with 10,000 repetitions to determine the significance of each correlation. In each permutation, the observations for each species were shuffled, such that the ratio of presences to absences was preserved for each species, but were randomized with respect to time and site. A null distribution of correlations was estimated based on correlations calculated for the permuted data. Correlations which were significantly larger in magnitude than expected by chance, for an overall false discovery rate of 0.05 (using the Benjamini-Hochberg FDR correction^62^), were kept in the network as interactions.

### Null Models

Two null models were used to assess comparative fit of the network models. The first null model was a weighted coin flip for each species. That is, the presence or absence of a species at any time point was predicted by the probability of presence in the training set. This null model captured weighted presence/absence probabilities, but ignored the time component of the data.

The second null model was a disconnected DBN, where the probability that a species was present was independent of all others, conditional only on its own presence or absence in the previous time step. Essentially, the DBN structure was given by a network where each species connects only to itself. The parameters of this model were calculated as for the full DBN model. This null model captures the autocorrelation of the data, but no interactions between species.

### Model Comparison

The similarity between empirical networks and model networks was measured using two standard machine learning measures, precision and recall. Precision measures the fraction of links identified by the model which are present in the empirical web, while recall measures the fraction of empirical links which are correctly identified by the model:

9 $${\rm{Precision}}=\frac{{\rm{true}}\,{\rm{positives}}}{{\rm{true}}\,{\rm{positives}}+{\rm{false}}\,{\rm{positives}}}$$

10 $${\rm{Recall}}=\frac{{\rm{true}}\,{\rm{positives}}}{{\rm{true}}\,{\rm{positives}}+{\rm{false}}\,{\rm{negatives}}}$$

If most of the interactions identified by a model are present in the empirical web, the model will have high precision. If a model is able to identify most of the interactions in the empirical web, it will have high recall. The ideal model would have both precision and recall near 1, but there is often a tradeoff between the two. For example, a model which predicts that all pairs of species interact would have high recall (it would identify all empirically present interactions), but low precision (it would falsely predict many empirically absent interactions). Conversely, a model which predicted only a single true positive would have high precision (all of its positives would be true positives), but low recall (it would fail to identify most empirically present interactions).

The significance of the precision and recall values was calculated using a permutation test. For each permutation, the model network was shuffled such that each species had the same number of incoming links, but the identity of those species was randomized. This constraint was added to replicate the DBN constraint on the number of parents, and the soft constraint of regularization in the Lasso model. A side effect of this permutation procedure is that although the number of true positives will vary by randomization, the denominators of the precision and recall formulas will not. Put another way, the precision and recall values will be perfectly correlated, such that the *p*-values for precision and recall of a given model will always be identical. Before calculating precision and recall, all interactions between a species and itself were excluded from both empirical and inferred networks. This is because these links represent cannibalism or self-regulation in the empirical networks, but represent autocorrelation in the inferred networks.

To compare model predictive performance, fit models were used to predict out of sample data. We calculated the log likelihood of each model correctly predicting the entire test dataset. For the Tatoosh dataset, separate models were fit to 14 cross-validation sets, resulting in 14 log likelihoods per model. The likelihood distributions of the models was compared to that of the disconnected DBN null model using a Wilcoxon rank sum test, with *p*-values corrected for a false discovery rate of 0.05 across all models.

### Data availability

Data used in this study can be found on Dryad at doi:10.5061/dryad.8m11n.

## Results

### DBN Convergence

Individual runs were relatively similar to the overall consensus networks, in both the France and Tatoosh datasets, suggesting that the algorithm was finding similar high-quality solutions in different runs. In the France dataset, *d* ranged from 1.05% to 10.47%, with an overall median difference of 4.46%, and a slight decrease in *d* as the maximum number of parents increased ($media{n}_{2}=\mathrm{6.50 \% }$, $media{n}_{3}=\mathrm{5.26 \% }$, $media{n}_{4}=\mathrm{2.90 \% }$, $media{n}_{5}=\mathrm{2.88 \% }$). In the Tatoosh dataset, *d* ranged from 0.00% to 11.20%, with an overall median difference of 4.67%. Median difference increased slightly with the maximum number of parents ($media{n}_{2}=\mathrm{3.14 \% }$, $media{n}_{3}=\mathrm{4.53 \% }$, $media{n}_{4}=\mathrm{5.61 \% }$, $media{n}_{5}=\mathrm{5.49 \% }$).

### Network Structures

All models inferred networks which were significantly related to empirical networks for one of the two datasets, but no model successfully inferred empirical networks for both. In the France dataset, only the networks inferred by Lasso models had higher precision and recall of the true trophic network than expected by chance (Table 1). The 2-parent DBN model was marginally significant (*p* = 0.06). The Pearson’s correlation coefficient network had very high recall (0.90) but relatively low precision (0.19), suggesting that although the correlation network had many interactions (${\rm{connectance}}\,\mathrm{=\; 0.85}$), the interactions had little relation to the trophic network structure.

**Table 1:** Precision and recall of model networks compared to empirical network structures for the French stream trophic network, Tatoosh trophic network, and Tatoosh nontrophic network.

Model	France		Tatoosh Trophic		Tatoosh Nontrophic	
	Precision	Recall	Precision	Recall	Precision	Recall
DBN-2	0.33	0.060	0.24	0.016	0.31^*^	0.027^*^
DBN-3	0.20	0.065	0.15	0.013	0.27	0.029
DBN-4	0.22	0.083	0.18	0.014	0.27	0.029
DBN-5	0.23	0.0898	0.20	0.016	0.27	0.029
Lasso-1st	0.24^*^	0.571^*^	0.19	0.193	0.22	0.297
Lasso-2nd	0.23^**^	0.685^**^	0.21	0.415	0.20	0.533
Pearson	0.19	0.905	0.18	0.155	0.25^***^	0.278^***^

Precision and recall values are given with significance based on a permutation test (<0.05: ^*^, <0.01: ^**^, <0.001: ^***^), with *p*-values corrected across all models and networks for an overall false discovery rate of $\alpha =0.05$.

**Figure 2:**
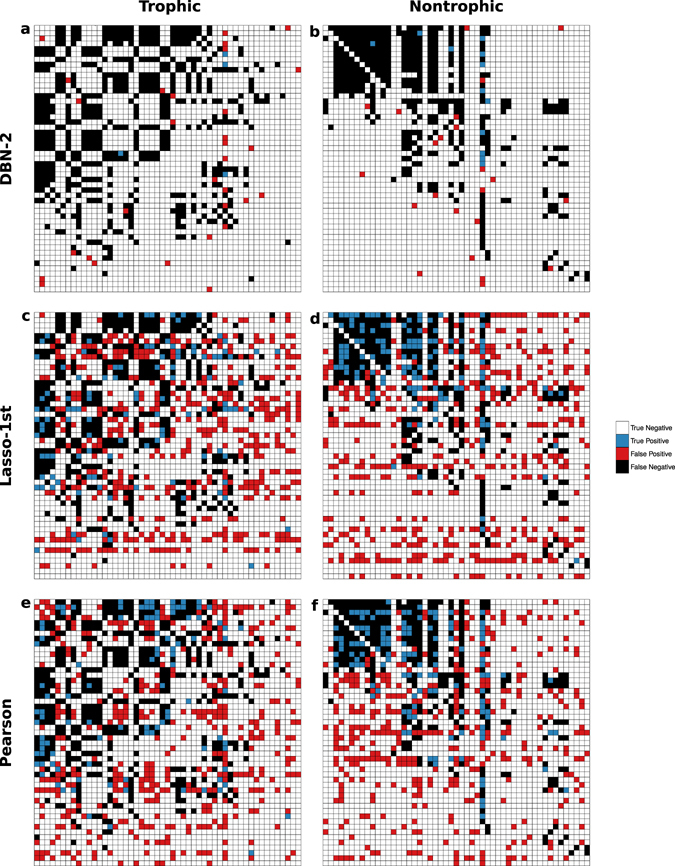
Structural comparison between empirical and model adjacency matrices for the Tatoosh system: (**a**) Trophic and DBN-2, (**b**) Nontrophic and DBN-2, (**c**) Trophic and Lasso-1st, (**d**) Nontrophic and Lasso-1st, (**e**) Trophic and Pearson, and (**f**) Nontrophic and Pearson. White boxes represent the absence of a link in both networks (true negative), blue represents a link present in both networks (true positive), black, a link present in the empirical but not model network (false negative), and red, a link present in the model but not empirical network (false positive).

All model networks for the Tatoosh trophic web performed poorly. No models produced a network which was significantly similar to the Tatoosh trophic network structure. Interestingly, the inferred networks were much more similar to the network of nontrophic interactions (Table 1, Fig. 2). The 2-parent DBN and Pearson networks were more similar to the nontrophic network than random (*p* = 0.02 for DBN-2, *p* < 0.01 for Pearson). The first-order Lasso (*p* = 0.10) and all other DBN networks (*p* = 0.06 for DBN-3, 4, and 5) were marginally significant (Table S1).

### Predictive Accuracy

**Figure 3:**
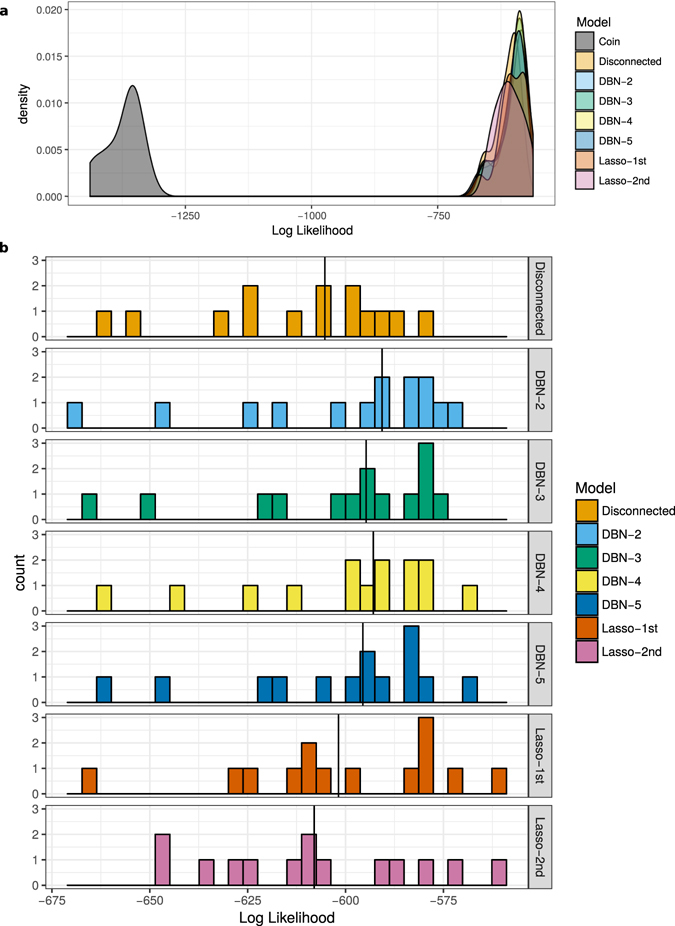
Log likelihood distributions for Tatoosh data subsamples, (**a**) including the weighted coin flip null model, and (**b**) excluding the weighted coin flip, and zooming in on the remaining models. Each observation represents the log likelihood of correctly predicting out-of-sample test data, given the model fit on one subsampled dataset. Black vertical lines in (**b**) represent the median log likelihoods for each model.

Both DBN and Lasso models predicted out-of-sample time points more accurately than null models. The disconnected DBN null model performed dramatically better than the coin flip null model in both datasets, suggesting that autocorrelation accounted for much of the trend in the data. In the France dataset, Lasso models performed better than null models, and similarly to each other. DBN models performed better than both Lasso and null models (Table 2). Due to the limited size of the Tatoosh dataset, models were trained on 14 data subsamples, each of which was used to predict the remaining out-of-sample points. This resulted in a distribution of likelihood values, rather than a single number (Fig. 3, Table S2). Although there was some overlap between the likelihood distributions (with the exception of the coin flip null), Wilcoxon signed-rank tests between the models and the disconnected null found some meaningful differences. The DBN models all performed better than the disconnected model (*p* = 0.003, 0.003, <0.001, and 0.001 for 2, 3, 4, and 5 max parents, respectively). In contrast, neither Lasso model had a median that significantly differed from the disconnected null (*p* = 0.162 and 0.426 for the first and second-order models, respectively).

**Table 2:** Out-of-sample predictive accuracy for the French stream dataset.

Model	Log Likelihood	Median Probability
Coin	−26205.80	0.71
Disconnected	−11447.79	0.92
DBN-2	−10653.97	0.94
DBN-3	−10437.39	0.95
DBN-4	−10476.15	0.95
DBN-5	−10474.91	0.95
Lasso-1st	−11098.84	0.93
Lasso-2nd	−11017.07	0.94

Columns represent the model name, log likelihood of correctly predicting test data, and median probability of correctly predicting a single species at a single out-of-sample time point.

The predictive accuracy of DBN models did not appear to be limited by the constraint on the number of parents. In the France dataset, the DBN model with 2 parents performed worse than the others, but the model with 3 parents had the best predictive accuracy overall (Table 2). The 2 parent DBN performed best of all the Tatoosh models (Fig. 3).

## Discussion

Using three methods taken from microbial and gene network inference, we found mixed success for all methods. Although some inferred structures were significantly similar to empirical structures, precision and recall were relatively low overall, so even models which were significantly better than random were far from exact matches to the empirical structure. DBN-2 and Pearson significantly captured the nontrophic structure of the Tatoosh network, but only the Lasso regression variants significantly captured the French piscivory network. The models underperformed relative to a recent study of network inference methods applied to simulated abundance data^63^, suggesting either a general limitation of network inference using presence-absence data, or a limitation of simulations to capture the error and dynamics of empirical systems.

It is worth noting that many models are marginally significant for both the France and Tatoosh nontrophic networks. Although we should be cautious not to overstate marginally significant results, this does point to some level of generality in the performance of the models. The three methods are uneven in performance between the two datasets, but DBN and Lasso perform moderately well on both. A comparison of methods for gene network inference also found a good deal of variability in performance across datasets^20^, and no model was the best performer on all datasets. This resulted from the fact that each model had specific biases, which had more or less effect, depending on the specifics of the dataset. The models examined here also face different limitations and assumptions that affect their performance. The DBN model is computationally limited to be quite sparse, so in general it can be expected to have low recall (Table 1). The Lasso and Pearson methods tend to identify many more possible interactions, especially in the France piscivory system, which had more data. Milns *et al*.^28^ also found that the Lasso tended to overfit relative to Bayesian networks. Another limitation of the Lasso model is that it is fit one species at a time, and therefore, the regularization parameter will vary from species to species. Interestingly, this caused a “striped” pattern in the resulting networks (Fig. 2c and d), one which did not visibly reflect the true structure of the network. An approach to overcoming the limitations of the individual models is to combine the results of several methods, which was found to improve the overall performance of network inference models on several benchmark gene regulation networks^20^. This is a promising future avenue of research in ecological network inference.

Interestingly, some models capture the trophic structure of the France piscivory network, but no models capture the trophic structure of Tatoosh, although they perform relatively well on the nontrophic structure. Unfortunately, not enough information was available to construct a reliable nontrophic network for the France system, so we can only speculate if these methods are better at capturing nontrophic information in general, or if there is something specific to the Tatoosh system or dataset. Co-occurrence networks are generally used to infer competitive interactions specifically^24, 63, 65^, although co-occurrence modelling has also been used to incorporate the effect of both trophic and nontrophic interactions into species distribution models^66–68^. Because we have data on the dynamics of the system through time, we might naively expect to identify the effects of both trophic and nontrophic interactions, and at least in the French network, this was the case. However, it is possible that presence-absence data is too blunt an instrument to capture predator-prey dynamics in some systems. Simple Lotka-Volterra predator-prey dynamics are neutrally stable^69^, so predator-prey pairs which do not quickly drive each other to extinction would be expected to persist for long periods of time. This means that in many cases, both predator and prey would simply appear as present for long periods of time. In contrast, competitive exclusion by definition results in the local extinction of one species, and therefore might be easier to capture in this type of data. In this way, the detection threshold of the data may influence the interactions the model can infer.

The many differences between these datasets allow us to see the variability in performance from the network inference algorithms, but make it difficult to identify specific factors underlying those differences. However, one likely factor is the type of competition dominant in each of these systems. In the Tatoosh system, competition for physical space strongly structures the system^40^. Most sessile species are either filter feeders or photosynthetic, and as a result, are directly competing for space on the rock or other individuals, rather than competing for a limited food resource. In this zone of the intertidal, bare rock is generally revealed only in response to disturbance, and is quickly colonized^40^. Competition for space in the Tatoosh middle intertidal is so strong that competitive interactions may drown out the signal of feeding interactions (but see ref. 70). In contrast, physical space in rivers is less limiting, so feeding interactions and competition for shared prey are more likely to be strong drivers in the French piscivory network.

Despite their mixed success for predicting network structures, the DBN and Lasso models were relatively good at predicting short term dynamics. The DBN models were consistently predictive in both the France and Tatoosh systems, and although the Lasso models were not as predictive of the Tatoosh system, they performed well on the France dataset. DBNs and Lasso are fundamentally predictive models, with the network structures produced more as a side effect than an end in itself. These models are clearly effectively finding predictive patterns in the data; however, these patterns may not always correspond to ecological interactions. Even in the model networks which corresponded well to the empirical networks, there were many false positives and false negatives. False negatives are not especially surprising, since empirical networks are likely to contain relatively unimportant interactions which may not have much influence on the community dynamics. The false positives demonstrate that species were predicting each other’s dynamics without directly interacting. Some of these predicted interactions likely result from indirect effects: causal effects that occur without direct interaction between the two species. Indirect effects are known to drive dynamics in the Tatoosh intertidal (*e.g*. refs 70–73). False positives might also occur when two species share a common driver, as has been found in other co-occurrence studies^74^. In the lagged models used in this study, species which share a predator, resource or environmental stressor might be inferred to “interact” if they respond at different time scales. The species which responds at close to a lag-1 time scale would, for example, predict a species which responds at close to a lag-2 time scale. An interesting extension of this work would be to consider how the choice of lag affects the structure of the inferred network.

Although none of these methods was consistently successful, they are worth exploring further, both for different systems and different types of data (for example, abundance rather than presence-absence data). It would also be valuable to other approaches to presence-absence co-occurrence modelling.

No method is a strictly significant predictor of network structure for both systems, but it is clear that methods like these are capable of capturing some dynamical signal of the underlying interactions. This suggests that they could reasonably be used in microbial systems, but only with great caution; for these two datasets, even the model networks which significantly match empirical structures had relatively low precision and recall. In the absence of known microbial networks^16^, data for macroscopic communities can provide a useful proxy for validating the use and interpretation of network inference methods. As machine learning and correlational methods continue to grow in popularity, it is vital that we validate their results, and consider what we can and cannot learn from the networks they produce.

## Electronic supplementary material

Supplemental Information for: Ecological Network Inference From Long-Term Presence-Absence Data
