# Supplementary material for: Ecological Network Inference From Long-Term Presence-Absence Data
Source: Sci Rep. 2017 Aug 2;7:7154. doi: 10.1038/s41598-017-07009-x (PMC5541006; doi:10.1038/s41598-017-07009-x)
Supplement: Supplementary file 1 — Supplemental Information for: Ecological Network Inference From Long-Term Presence-Absence Data [file 41598_2017_7009_MOESM1_ESM.pdf]

Supplemental Information for: *Ecological Network Inference From Long-Term Presence-Absence Data*

Elizabeth L. Sander[1,*], J. Timothy Wootton[1], and Stefano Allesina[1,2]

[1]University of Chicago, Department of Ecology and Evolution, Chicago, 60637, USA
[2]University of Chicago, Computation Institute, Chicago, 60637, USA
[*]esander@uchicago.edu

Model	Subsample	Median Probability	Log Likelihood
Coin	1	0.71	-1351.65
Coin	2	0.71	-1438.84
Coin	3	0.72	-1359.96
Coin	4	0.71	-1408.46
Coin	5	0.72	-1324.23
Coin	6	0.71	-1349.12
Coin	7	0.71	-1347.53
Coin	8	0.71	-1363.43
Coin	9	0.71	-1366.17
Coin	10	0.71	-1355.30
Coin	11	0.71	-1432.69
Coin	12	0.71	-1399.75
Coin	13	0.71	-1337.17
Coin	14	0.72	-1387.19
Disconnected	1	0.81	-581.15
Disconnected	2	0.79	-652.67
Disconnected	3	0.81	-630.82
Disconnected	4	0.80	-662.79
Disconnected	5	0.82	-588.37
Disconnected	6	0.81	-604.36
Disconnected	7	0.81	-598.80
Disconnected	8	0.81	-591.53
Disconnected	9	0.82	-594.54
Disconnected	10	0.81	-598.15
Disconnected	11	0.81	-624.40
Disconnected	12	0.80	-613.72
Disconnected	13	0.82	-606.19
Disconnected	14	0.81	-623.79
DBN-2	1	0.83	-571.70
DBN-2	2	0.82	-646.60
DBN-2	3	0.82	-617.19
DBN-2	4	0.82	-669.52
DBN-2	5	0.84	-582.59
DBN-2	6	0.84	-590.07
DBN-2	7	0.84	-579.43
DBN-2	8	0.83	-584.08
DBN-2	9	0.83	-591.26
DBN-2	10	0.84	-576.44
DBN-2	11	0.83	-594.09
DBN-2	12	0.82	-603.52
DBN-2	13	0.84	-579.63
DBN-2	14	0.83	-624.11

DBN-3	1	0.83	-571.70
DBN-3	2	0.82	-646.60
DBN-3	3	0.82	-617.19
DBN-3	4	0.82	-669.52
DBN-3	5	0.84	-582.59
DBN-3	6	0.84	-590.07
DBN-3	7	0.84	-579.43
DBN-3	8	0.83	-584.08
DBN-3	9	0.83	-591.26
DBN-3	10	0.84	-576.44
DBN-3	11	0.83	-594.09
DBN-3	12	0.82	-603.52
DBN-3	13	0.84	-579.63
DBN-3	14	0.83	-624.11
DBN-4	1	0.83	-571.70
DBN-4	2	0.82	-646.60
DBN-4	3	0.82	-617.19
DBN-4	4	0.82	-669.52
DBN-4	5	0.84	-582.59
DBN-4	6	0.84	-590.07
DBN-4	7	0.84	-579.43
DBN-4	8	0.83	-584.08
DBN-4	9	0.83	-591.26
DBN-4	10	0.84	-576.44
DBN-4	11	0.83	-594.09
DBN-4	12	0.82	-603.52
DBN-4	13	0.84	-579.63
DBN-4	14	0.83	-624.11
DBN-5	1	0.83	-571.70
DBN-5	2	0.82	-646.60
DBN-5	3	0.82	-617.19
DBN-5	4	0.82	-669.52
DBN-5	5	0.84	-582.59
DBN-5	6	0.84	-590.07
DBN-5	7	0.84	-579.43
DBN-5	8	0.83	-584.08
DBN-5	9	0.83	-591.26
DBN-5	10	0.84	-576.44
DBN-5	11	0.83	-594.09
DBN-5	12	0.82	-603.52
DBN-5	13	0.84	-579.63
DBN-5	14	0.83	-624.11
Lasso-1st	1	0.71	-580.27
Lasso-1st	2	0.71	-609.93
Lasso-1st	3	0.73	-580.36
Lasso-1st	4	0.71	-607.62
Lasso-1st	5	0.72	-571.27
Lasso-1st	6	0.71	-605.50
Lasso-1st	7	0.73	-581.86
Lasso-1st	8	0.71	-562.52
Lasso-1st	9	0.71	-580.16
Lasso-1st	10	0.70	-622.67
Lasso-1st	11	0.70	-666.94
Lasso-1st	12	0.71	-629.32
Lasso-1st	13	0.72	-598.10

Lasso-1st	14	0.74	-612.47
Lasso-2nd	1	0.71	-580.27
Lasso-2nd	2	0.71	-609.93
Lasso-2nd	3	0.73	-580.36
Lasso-2nd	4	0.71	-607.62
Lasso-2nd	5	0.72	-571.27
Lasso-2nd	6	0.71	-605.50
Lasso-2nd	7	0.73	-581.86
Lasso-2nd	8	0.71	-562.52
Lasso-2nd	9	0.71	-580.16
Lasso-2nd	10	0.70	-622.67
Lasso-2nd	11	0.70	-666.94
Lasso-2nd	12	0.71	-629.32
Lasso-2nd	13	0.72	-598.10
Lasso-2nd	14	0.74	-612.47

Table S2. Out-of-sample prediction accuracy for different subsamples of the Tatoosh dataset. Columns represent the model, subsample number, the median probability of correctly predicting a single out-of-sample species at one time point, and the log likelihood of correctly predicting all species at all time points, given the model trained on on that data subsample. The log likelihood values are shown visually in main text Fig. 2.

Predator	Prey	Ref
Ameiurus melas	Cyprinus carpio	1
Ameiurus melas	Gymnocephalus cernua	2
Ameiurus melas	Lepomis gibbosus	1
Anguilla anguilla	Abramis brama	3, 4
Anguilla anguilla	Barbatula barbatula	5, 6
Anguilla anguilla	Cottus gobio	6, 7
Anguilla anguilla	Gasterosteus aculeatus	4, 6, 8
Anguilla anguilla	Gobio gobio	5, 6
Anguilla anguilla	Gymnocephalus cernua	3, 4, 9–11
Anguilla anguilla	Leuciscus leuciscus	3, 5
Anguilla anguilla	Lota lota	3
Anguilla anguilla	Phoxinus phoxinus	6, 8, 12, 13
Anguilla anguilla	Perca fluviatilis	3, 4, 11, 12
Anguilla anguilla	Rutilus rutilus	3–5, 8, 11
Anguilla anguilla	Sander lucioperca	4, 11
Anguilla anguilla	Salmo salar	14
Anguilla anguilla	Squalius cephalus	3
Blicca bjoerkna	Gymnocephalus cernua	10
Cottus gobio	Perca fluviatilis	15
Cottus gobio	Salmo trutta	16
Esox lucius	Abramis brama	17, 18
Esox lucius	Alburnus alburnus	17, 19–21
Esox lucius	Ameiurus melas	2, 22
Esox lucius	Anguilla anguilla	6, 18, 19, 21, 23, 24
Esox lucius	Barbatula barbatula	6, 7, 18, 19, 21, 25
Esox lucius	Cottus gobio	6, 18, 19, 21, 25
Esox lucius	Cyprinus carpio	26–28
Esox lucius	Gasterosteus aculeatus	6, 7, 17, 18, 21, 23–25, 29
Esox lucius	Gobio gobio	6, 7, 18, 19, 21, 27, 28, 30, 31
Esox lucius	Gymnocephalus cernua	2, 10, 20, 25, 32
Esox lucius	Lepomis gibbosus	27, 33, 34
Esox lucius	Leuciscus leuciscus	19, 21

Esox lucius	Lota lota	17, 35, 36
Esox lucius	Perca fluviatilis	17, 20, 23–25, 29, 35, 36
Esox lucius	Phoxinus phoxinus	6, 7, 17–19, 21, 23, 24, 29, 36
Esox lucius	Pungitius pungitius	25, 36, 37
Esox lucius	Rutilus rutilus	6, 7, 17–21, 24
Esox lucius	Sander lucioperca	20
Esox lucius	Salmo trutta	17, 19, 21, 23, 24, 28, 29, 36, 38, 39
Esox lucius	Salmo salar	14, 19, 24, 25
Esox lucius	Scardinius erythrophthalmus	32, 34
Esox lucius	Squalius cephalus	21, 30, 31
Esox lucius	Thymallus thymallus	19
Esox lucius	Tinca tinca	28, 30, 34
Gasterosteus aculeatus	Pungitius pungitius	40
Gymnocephalus cernua	Gymnocephalus cernua	10
Lota lota	Gymnocephalus cernua	2, 10
Lota lota	Esox lucius	41
Lota lota	Pungitius pungitius	36, 41
Lota lota	Salmo trutta	36, 42
Lota lota	Salmo salar	14
Lota lota	Sander lucioperca	43
Perca fluviatilis	Alburnus alburnus	44, 45
Perca fluviatilis	Gasterosteus aculeatus	18, 46
Perca fluviatilis	Gymnocephalus cernua	10, 47
Perca fluviatilis	Phoxinus phoxinus	46
Perca fluviatilis	Rutilus rutilus	18, 44, 45, 48–52
Perca fluviatilis	Sander lucioperca	47, 52, 53
Perca fluviatilis	Scardinius erythrophthalmus	45, 54
Perca fluviatilis	Squalius cephalus	45
Pungitius pungitius	Gasterosteus aculeatus	40
Salmo salar	Gasterosteus aculeatus	55, 56
Salmo trutta	Barbatula barbatula	57
Salmo trutta	Cottus gobio	58, 59
Salmo trutta	Gasterosteus aculeatus	9, 23, 60–62
Salmo trutta	Gobio gobio	6, 57, 63
Salmo trutta	Gasterosteus aculeatus	6, 61, 64, 65
Salmo trutta	Gymnocephalus cernua	66
Salmo trutta	Perca fluviatilis	23, 66, 67
Salmo trutta	Phoxinus phoxinus	13, 23, 61, 63, 68–70
Salmo trutta	Pungitius pungitius	36, 62, 66, 67
Salmo trutta	Rutilus rutilus	66
Salmo trutta	Salmo salar	14, 64, 65, 71, 72
Sander lucioperca	Alburnus alburnus	73–76
Sander lucioperca	Abramis brama	73–75, 77–79
Sander lucioperca	Cottus gobio	80
Sander lucioperca	Esox lucius	74
Sander lucioperca	Gymnocephalus cernua	10, 73–78, 81–84
Sander lucioperca	Lepomis gibbosus	73
Sander lucioperca	Perca fluviatilis	74, 76–85
Sander lucioperca	Rutilus rutilus	73, 74, 76–80, 82–85
Sander lucioperca	Salmo trutta	38
Squalius cephalus	Alburnoides bipunctatus	86
Squalius cephalus	Barbus meridionalis	86
Squalius cephalus	Cyprinus carpio	87

Table S3. Adjacency list of interactions in the French piscivory food web, with supporting references.

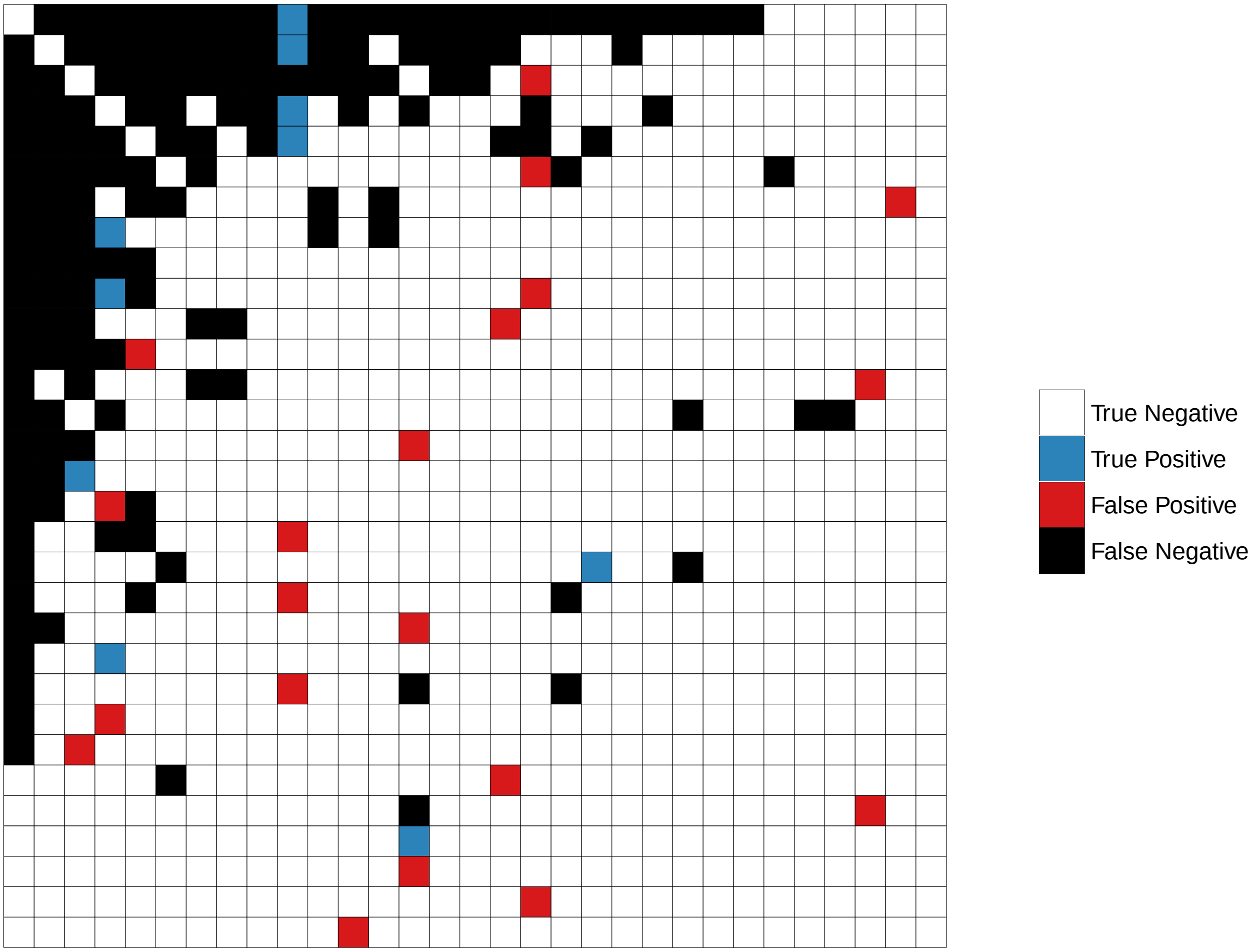

Figure S1. Structural comparison between empirical and model adjacency matrices as in Fig. 3a, but containing all species used in the DBN-2 model.

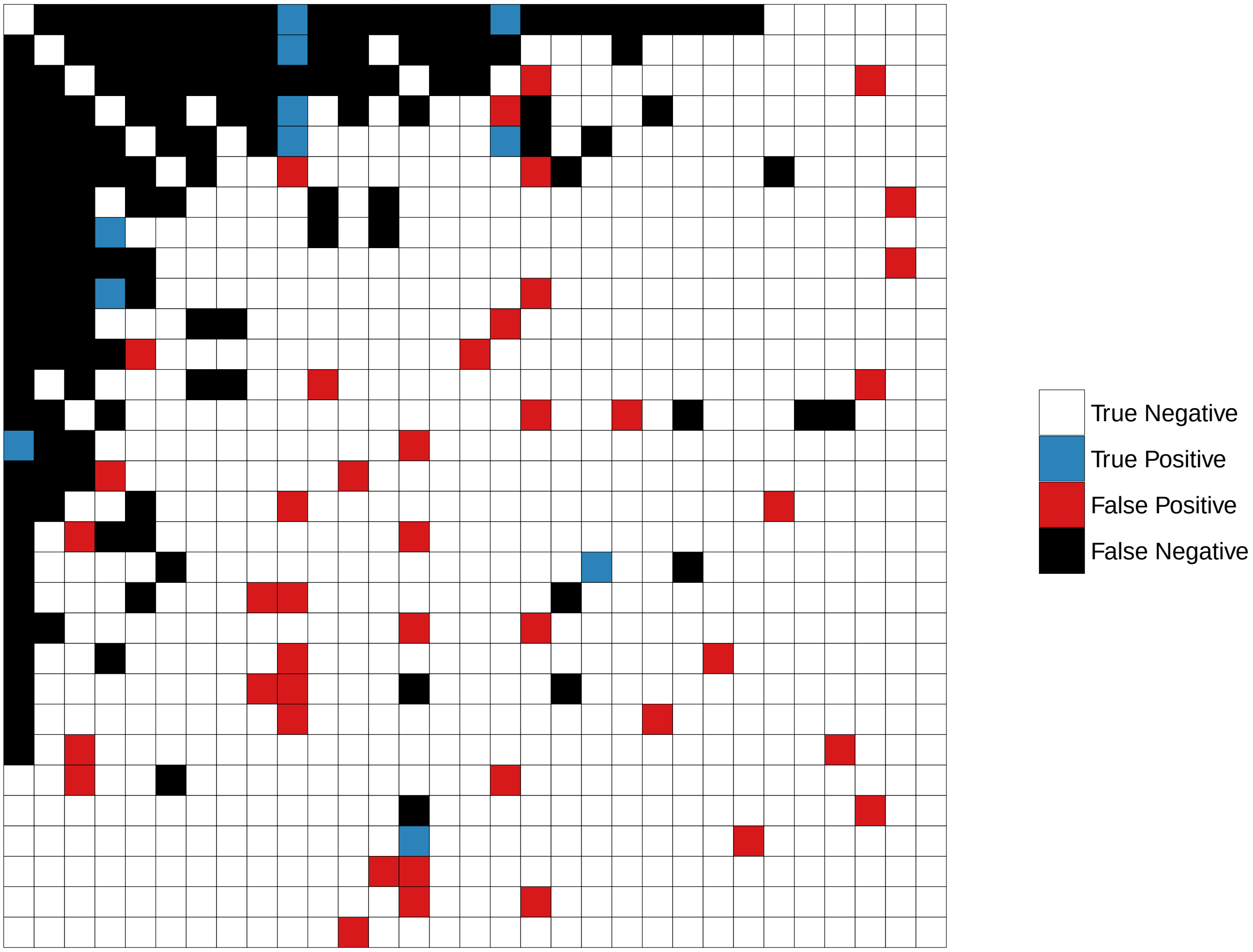

Figure S2. Structural comparison between empirical and model adjacency matrices as in Fig. 3, but for the France piscivory network and the DBN-3 model.

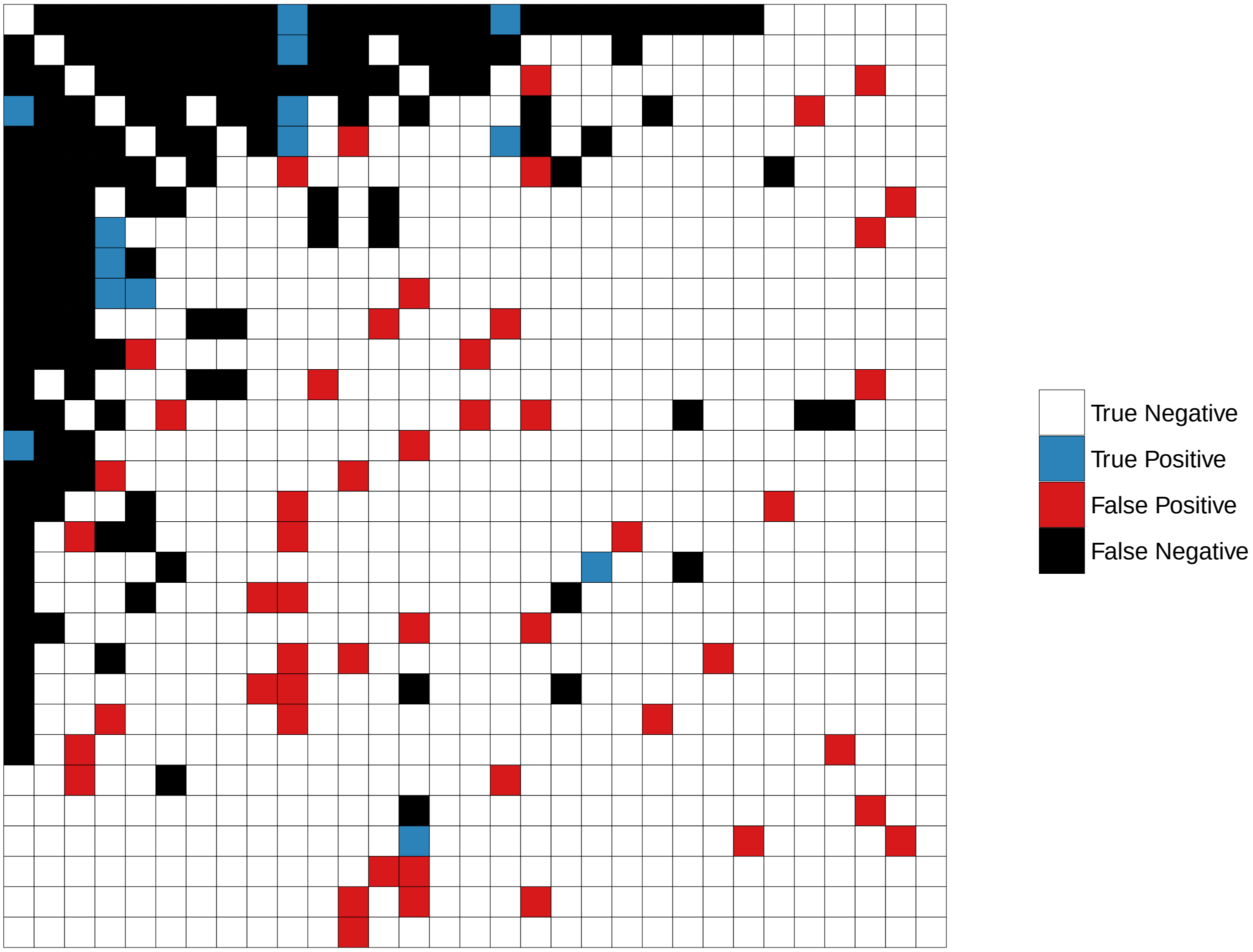

Figure S3. Structural comparison between empirical and model adjacency matrices as in Fig. 3, but for the France piscivory network and the DBN-4 model.

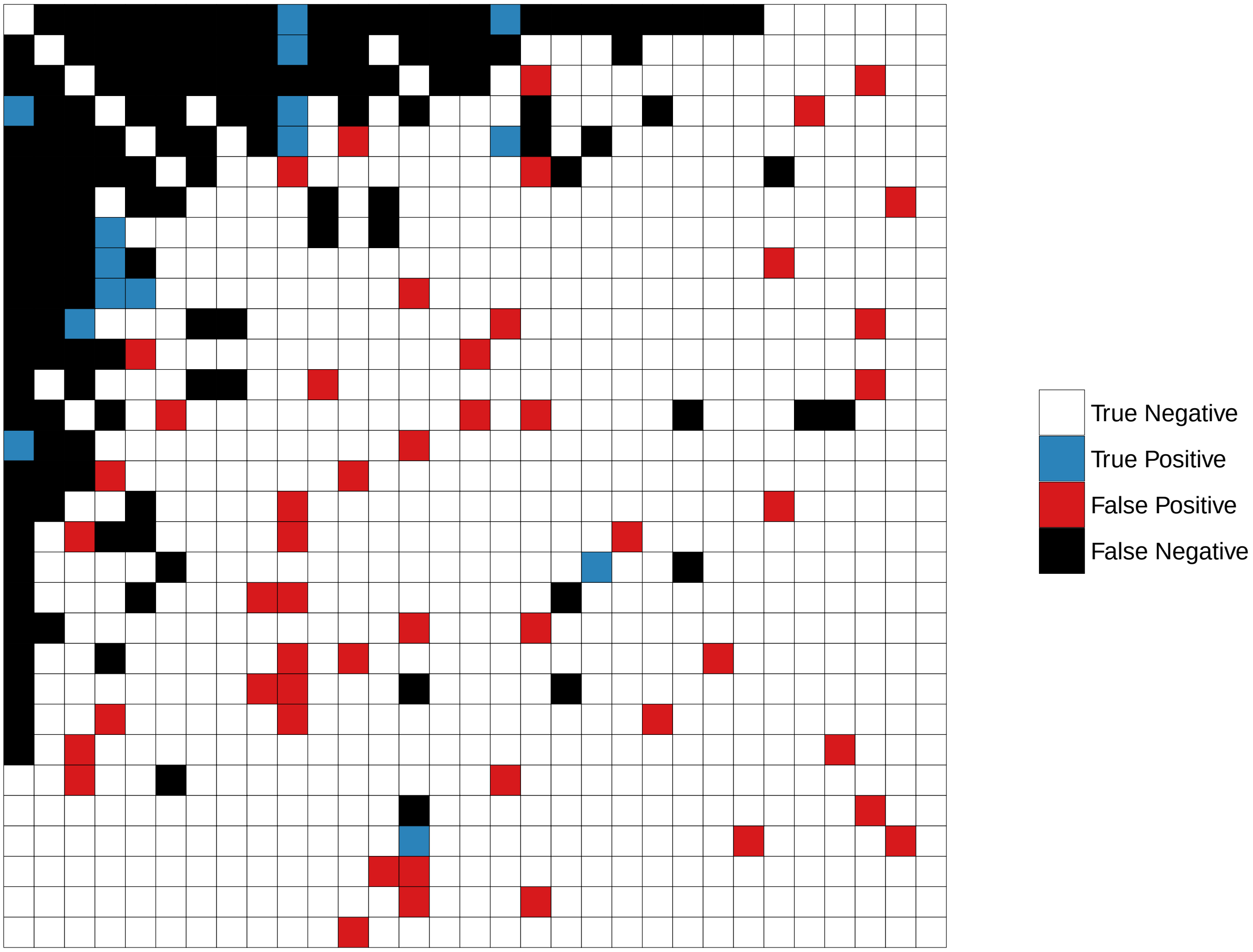

Figure S4. Structural comparison between empirical and model adjacency matrices as in Fig. 3, but for the France piscivory network and the DBN-5 model.

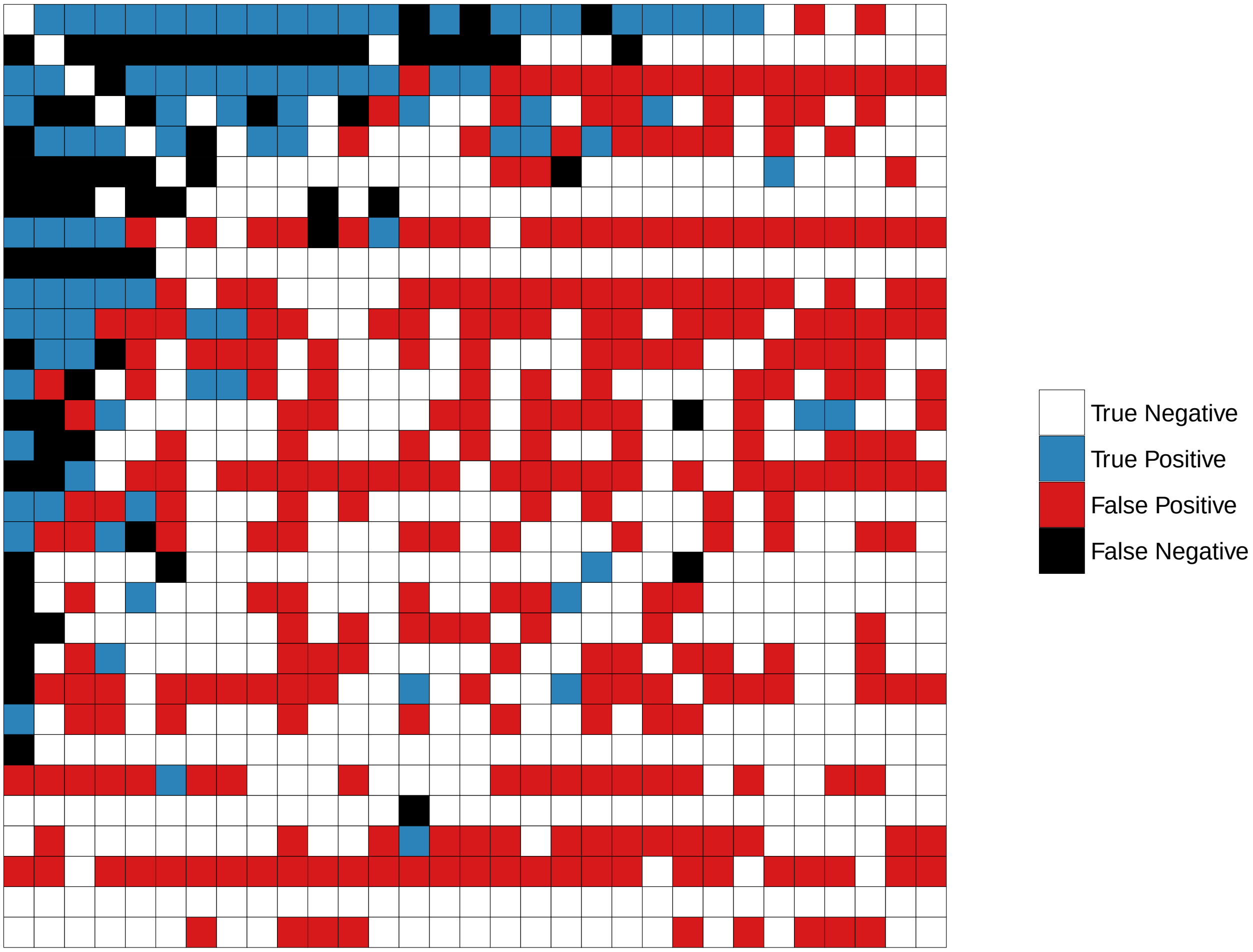

Figure S5. Structural comparison between empirical and model adjacency matrices as in Fig. 3, but for the France piscivory network and the Lasso-1st model.

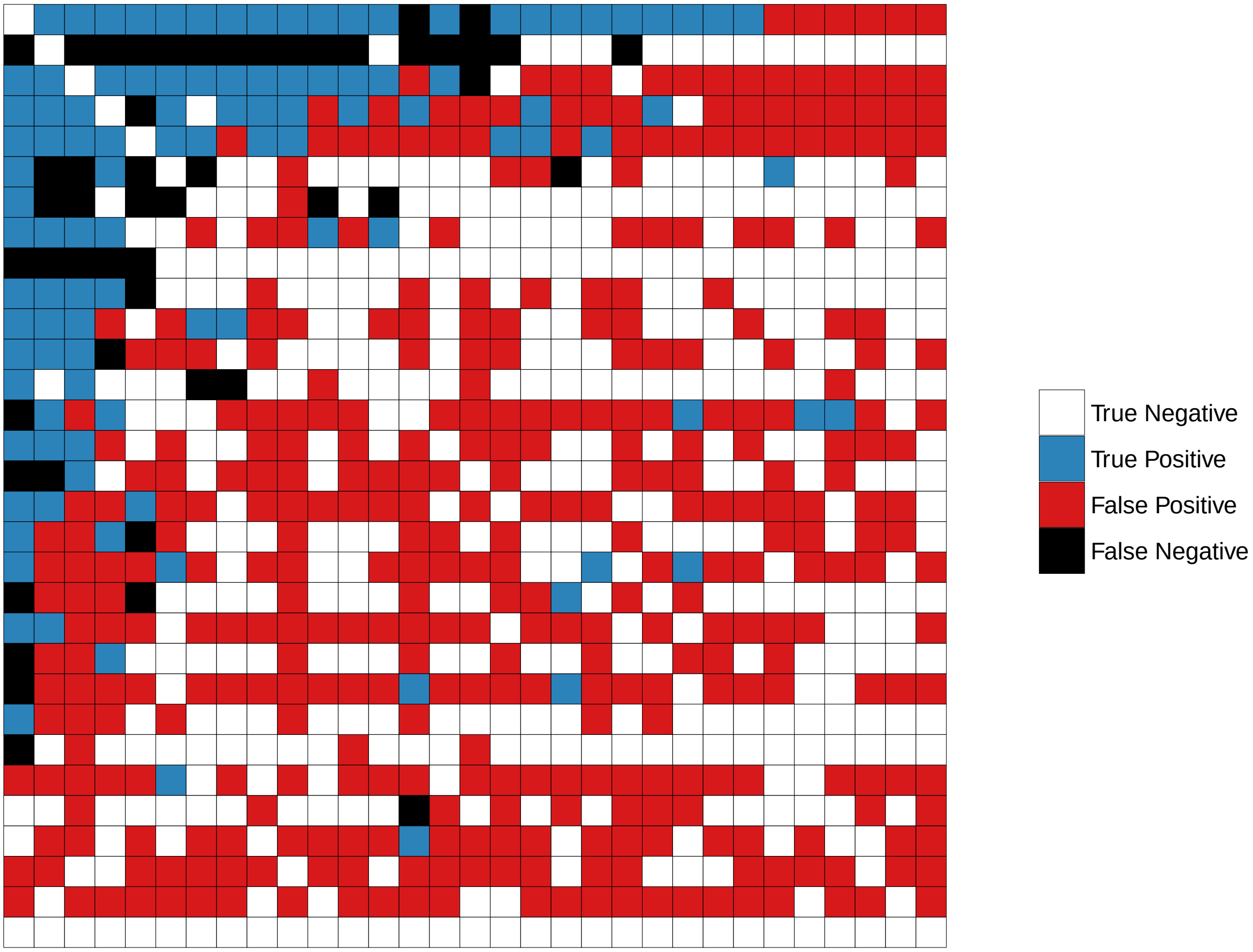

Figure S6. Structural comparison between empirical and model adjacency matrices as in Fig. 3, but for the France piscivory network and the Lasso-2nd model.

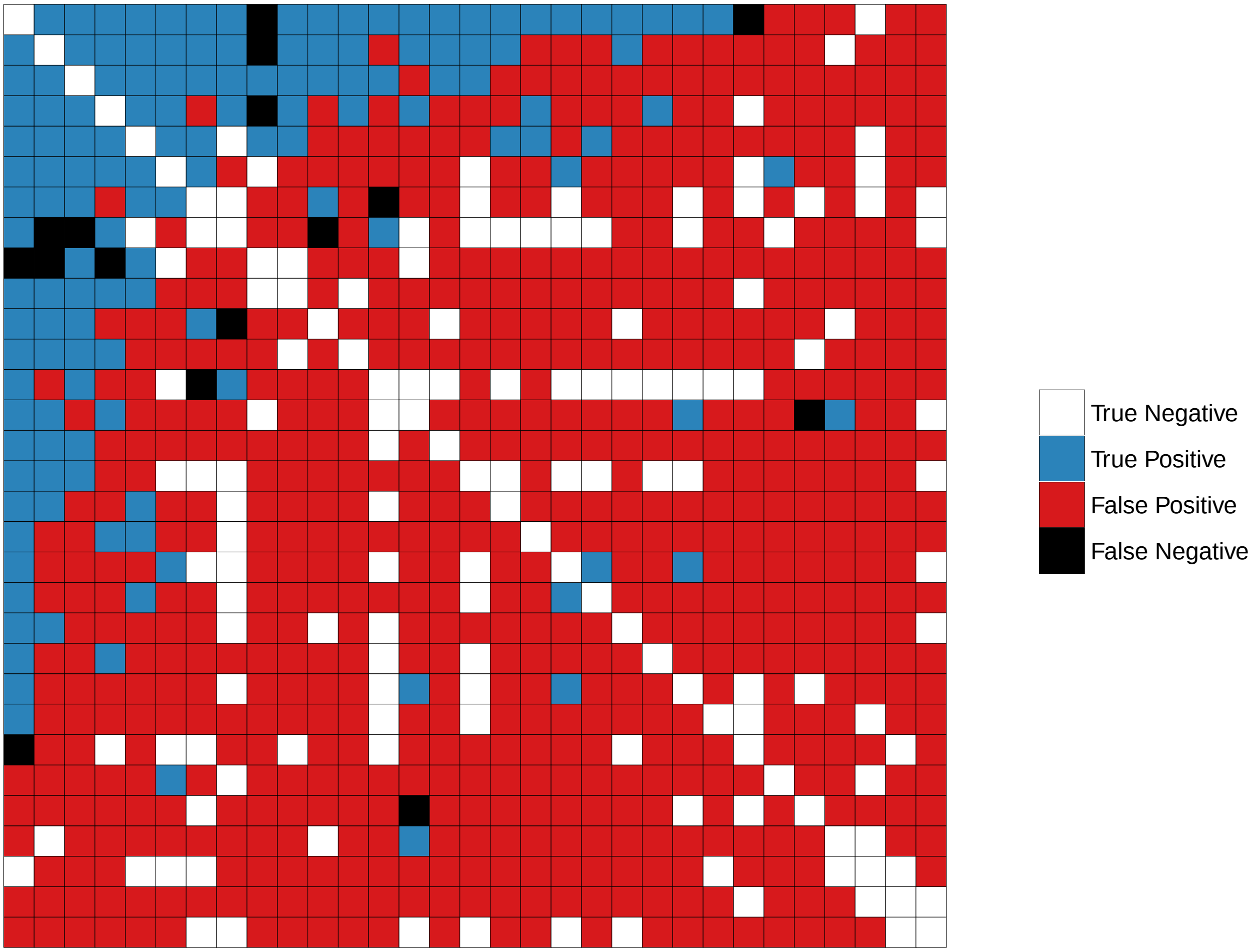

Figure S7. Structural comparison between empirical and model adjacency matrices as in Fig. 3, but for the France piscivory network and the Pearson model.

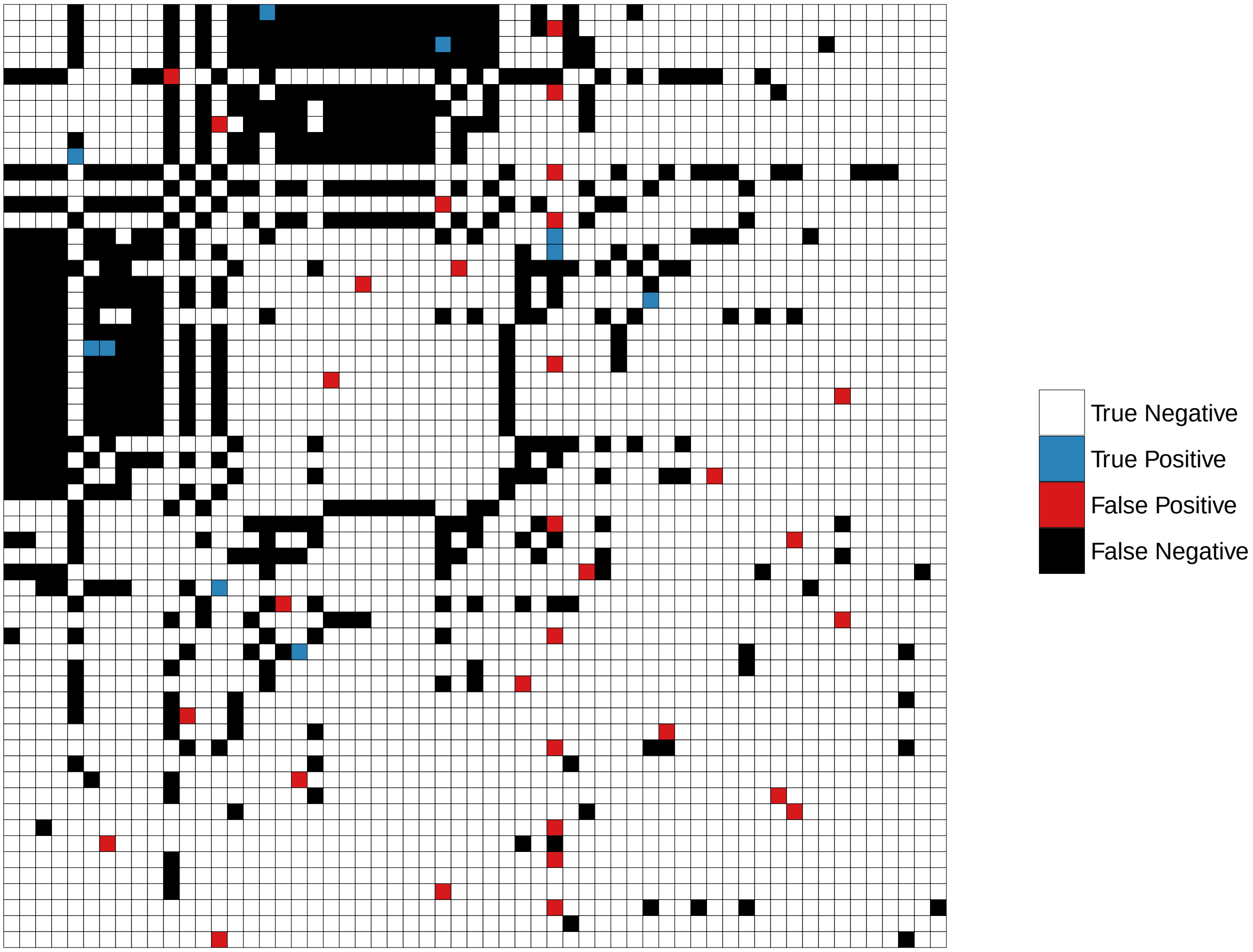

Figure S8. Structural comparison between empirical and model adjacency matrices as in Fig. 3, but for the Tatoosh trophic network and the DBN-2 model. Identical to Fig. 3a, except that it contains all species in the DBN and Pearson models, rather than excluding those which were excluded from the Lasso models.

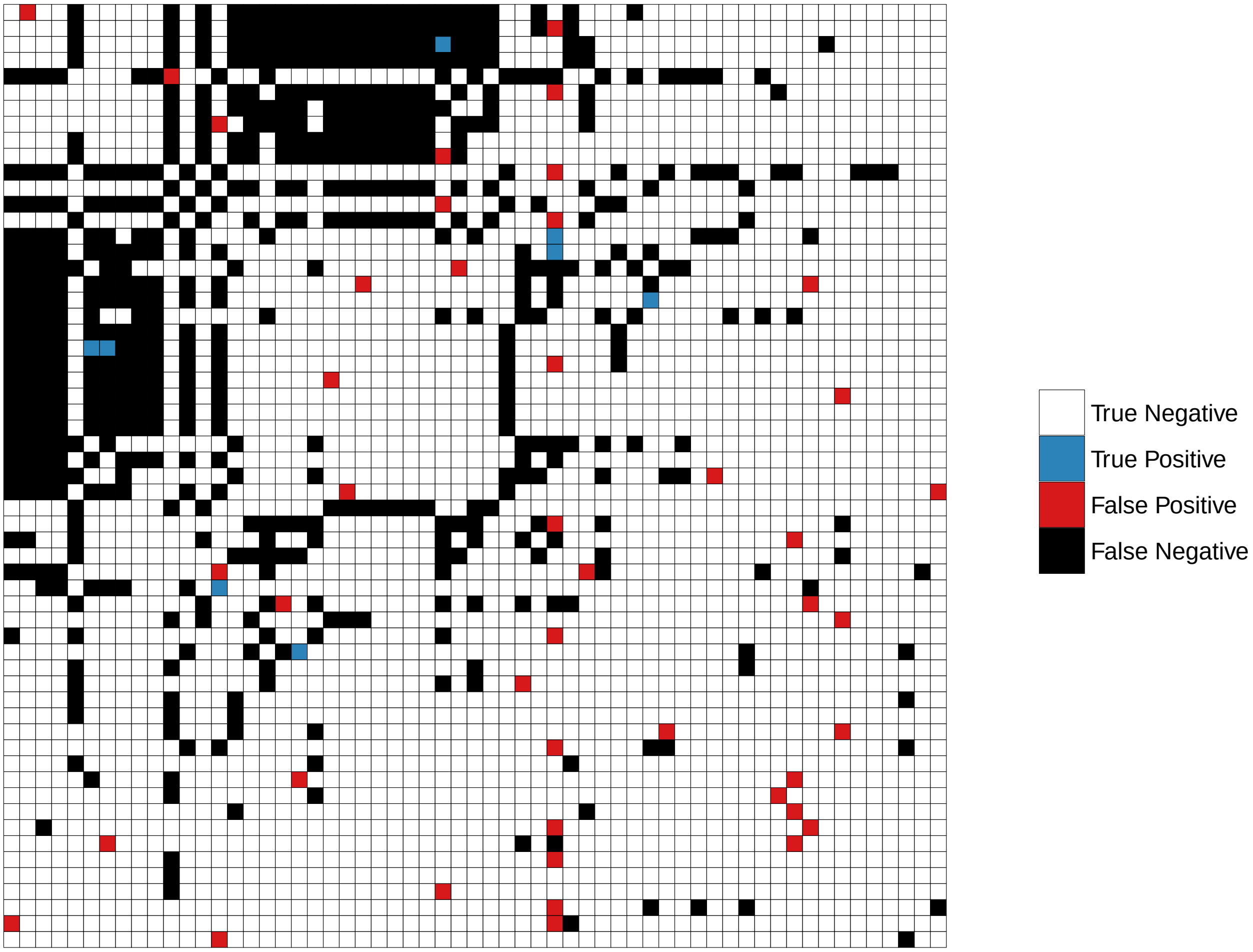

Figure S9. Structural comparison between empirical and model adjacency matrices as in Fig. 3, but for the Tatoosh trophic network and the DBN-3 model. Contains all species used in the DBN and Pearson models.

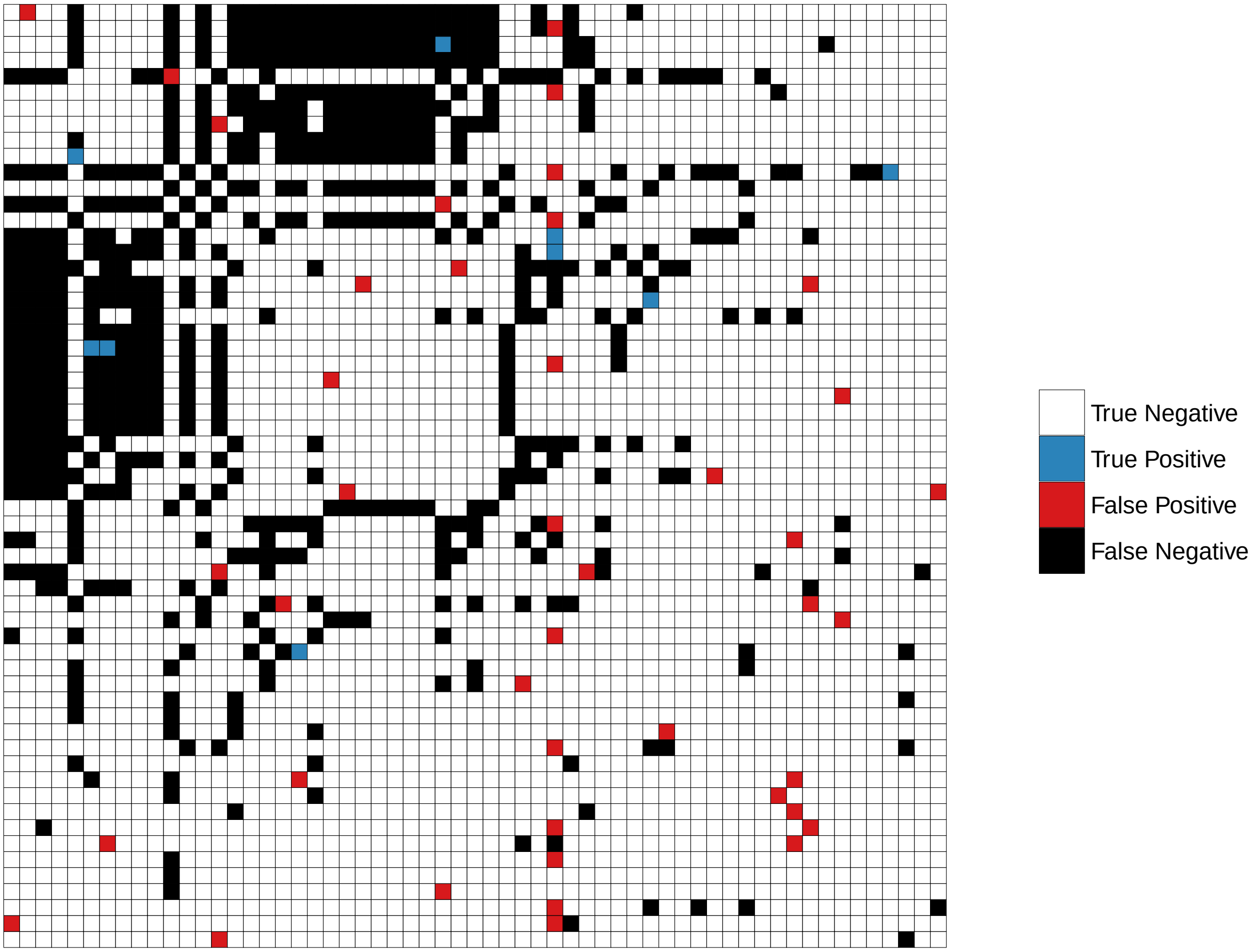

Figure S10. Structural comparison between empirical and model adjacency matrices as in Fig. 3, but for the Tatoosh trophic network and the DBN-4 model. Contains all species used in the DBN and Pearson models.

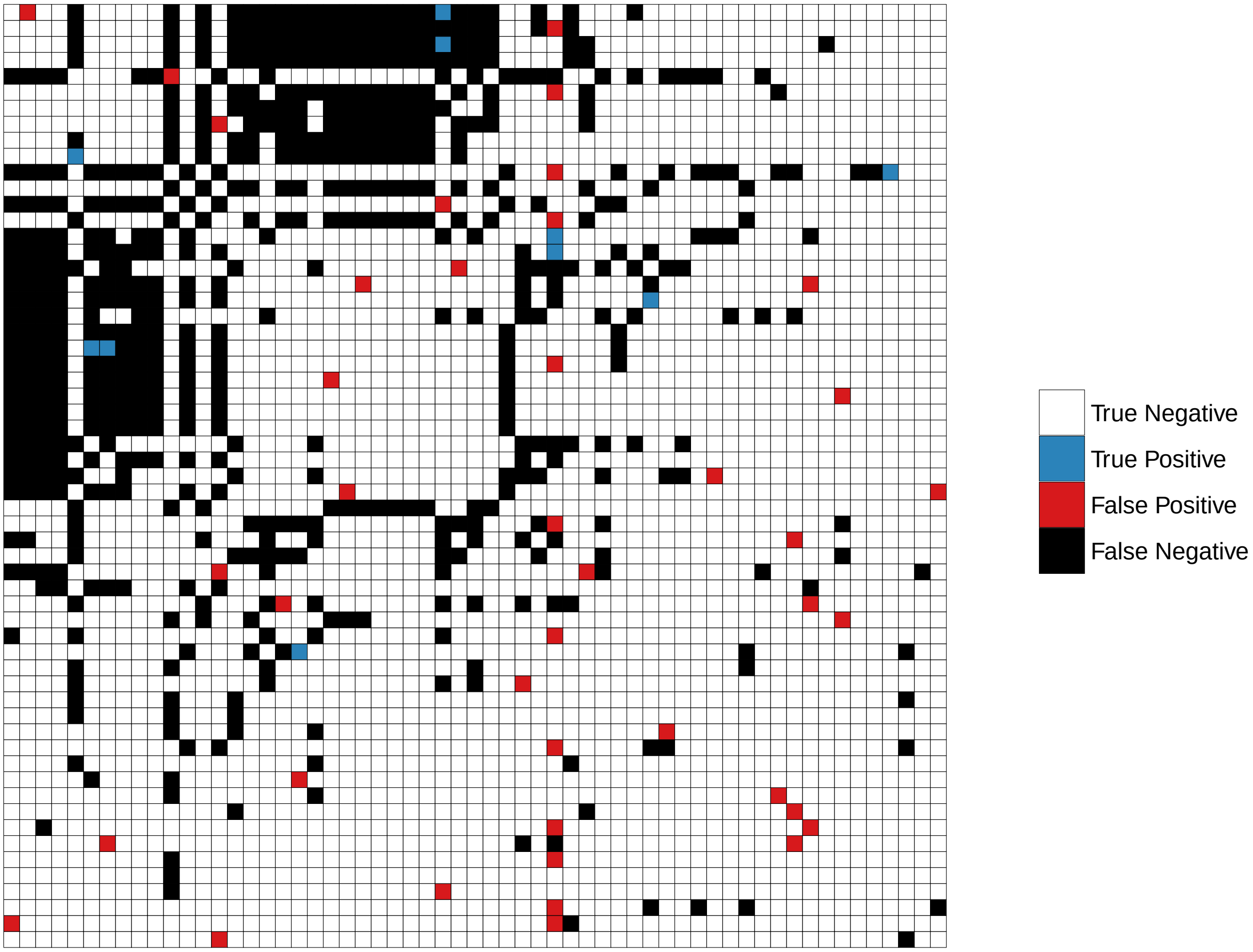

Figure S11. Structural comparison between empirical and model adjacency matrices as in Fig. 3, but for the Tatoosh trophic network and the DBN-5 model. Contains all species used in the DBN and Pearson models.

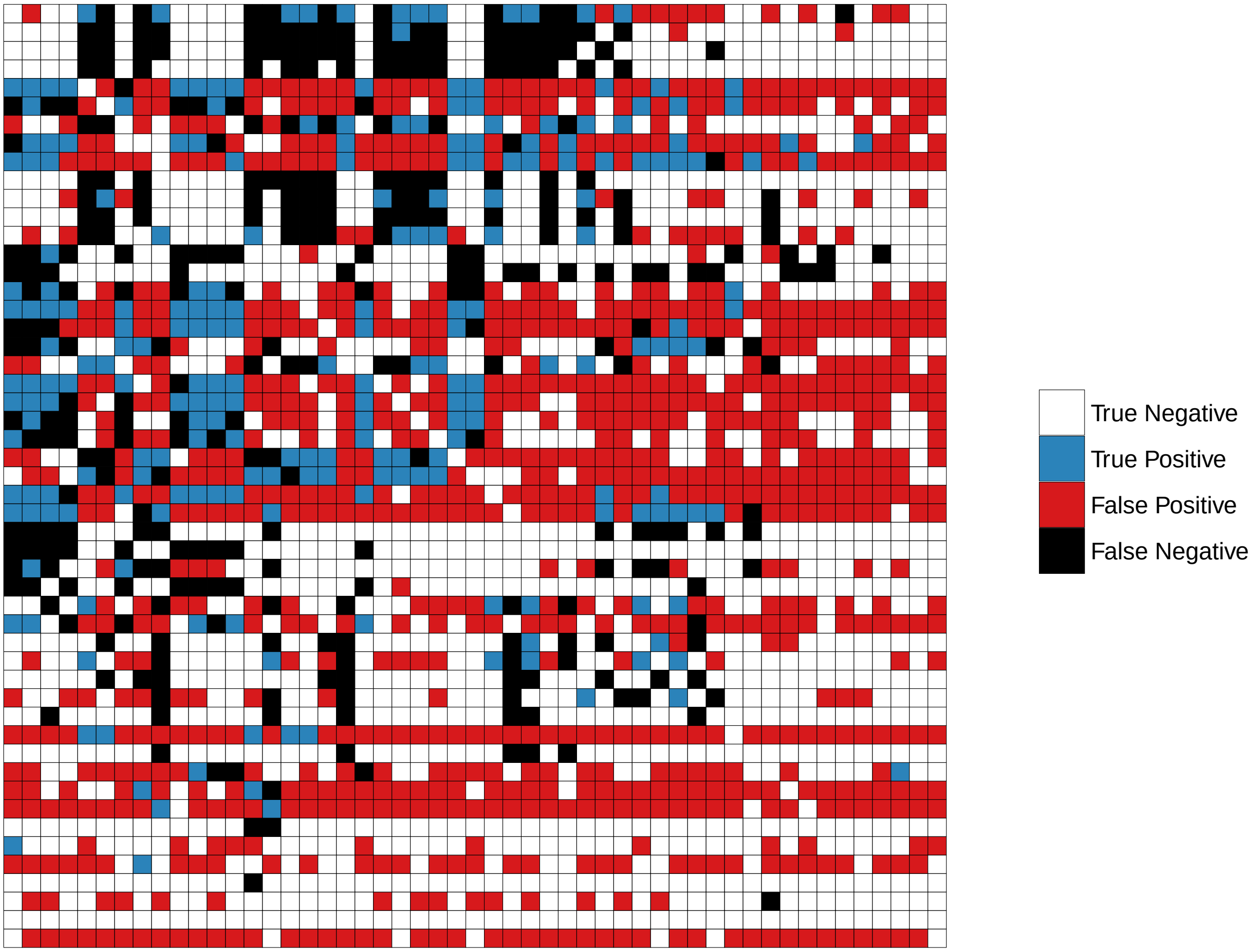

Figure S12. Structural comparison between empirical and model adjacency matrices as in Fig. 3, but for the Tatoosh trophic network and the Lasso-2nd model.

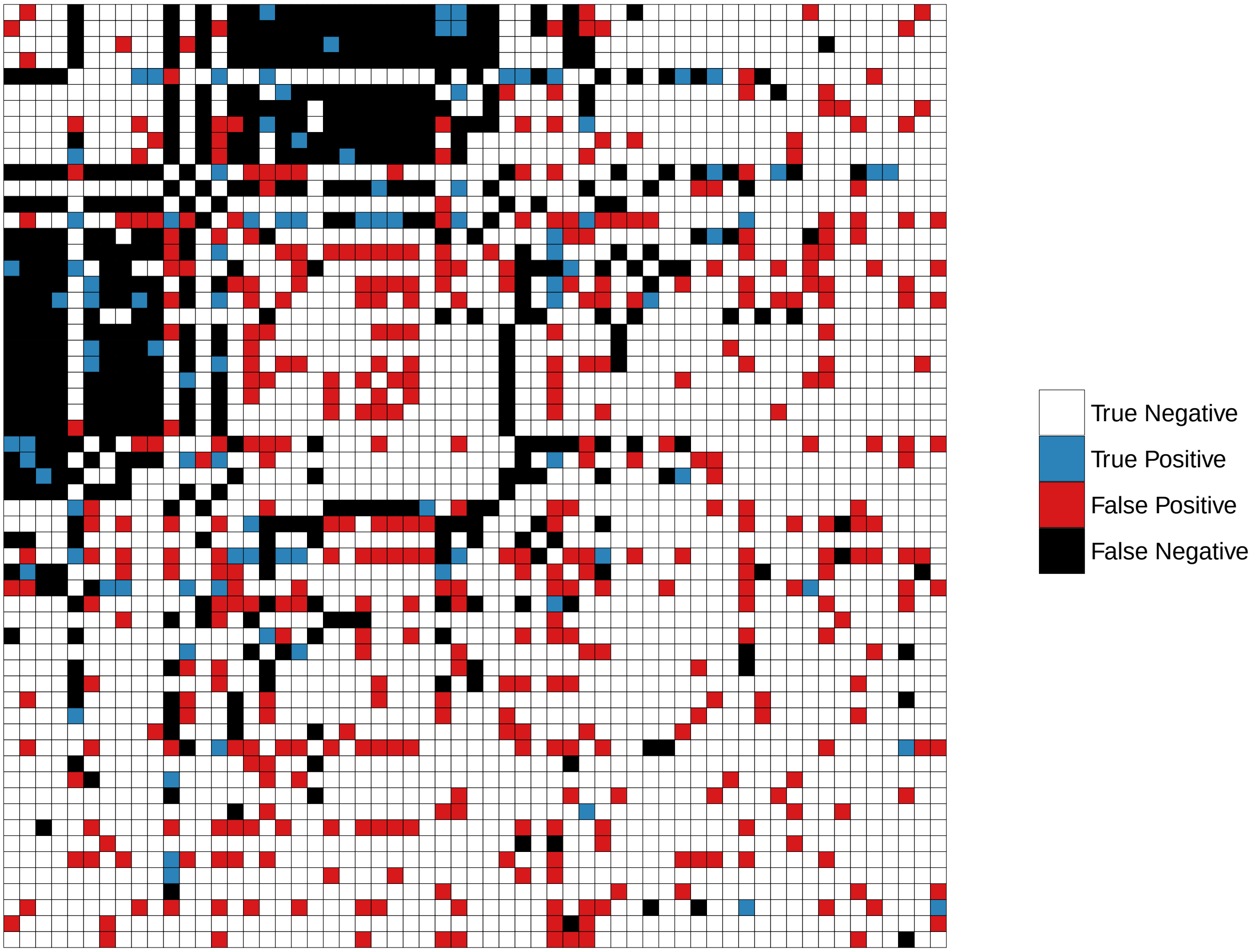

Figure S13. Structural comparison between empirical and model adjacency matrices as in Fig. 3, but for the Tatoosh trophic network and the Lasso-2nd model. Identical to Fig. 3e, except that it contains all species in the DBN and Pearson models, rather than excluding those which were excluded from the Lasso models.

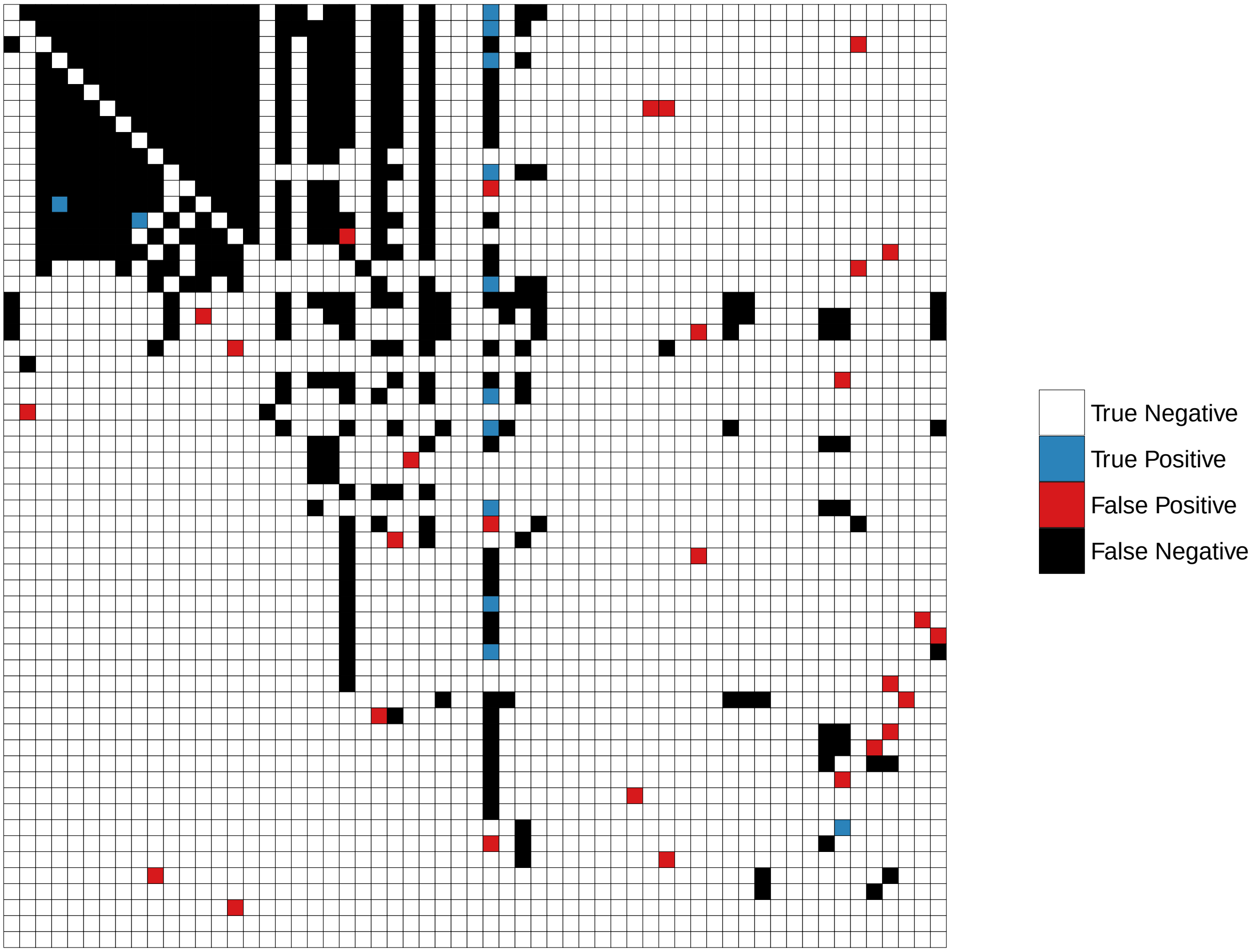

Figure S14. Structural comparison between empirical and model adjacency matrices as in Fig. 3, but for the Tatoosh nontrophic network and the DBN-2 model. Identical to Fig. 3b, except that it contains all species in the DBN and Pearson models, rather than excluding those which were excluded from the Lasso models.

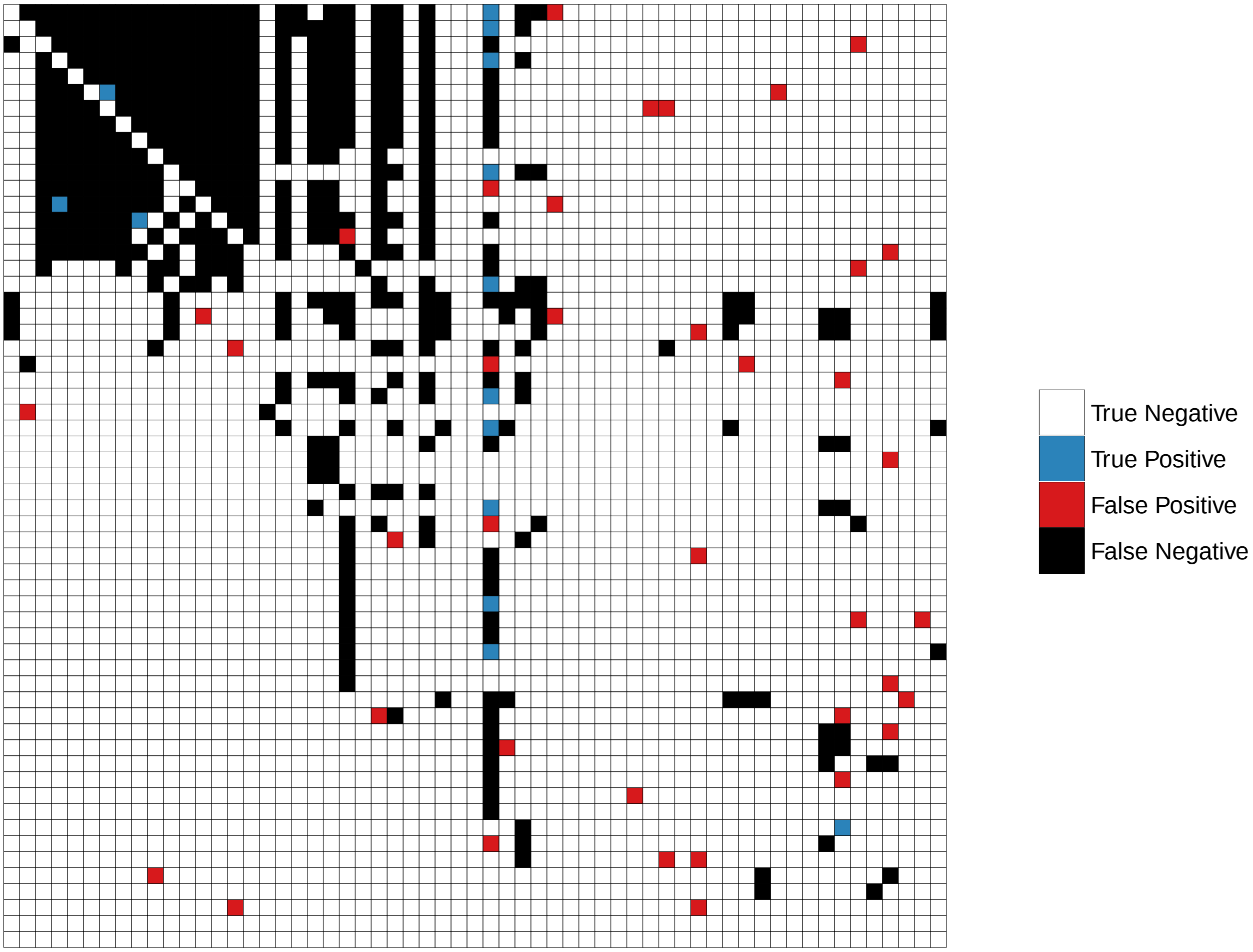

Figure S15. Structural comparison between empirical and model adjacency matrices as in Fig. 3, but for the Tatoosh nontrophic network and the DBN-3 model. Contains all species used in the DBN and Pearson models.

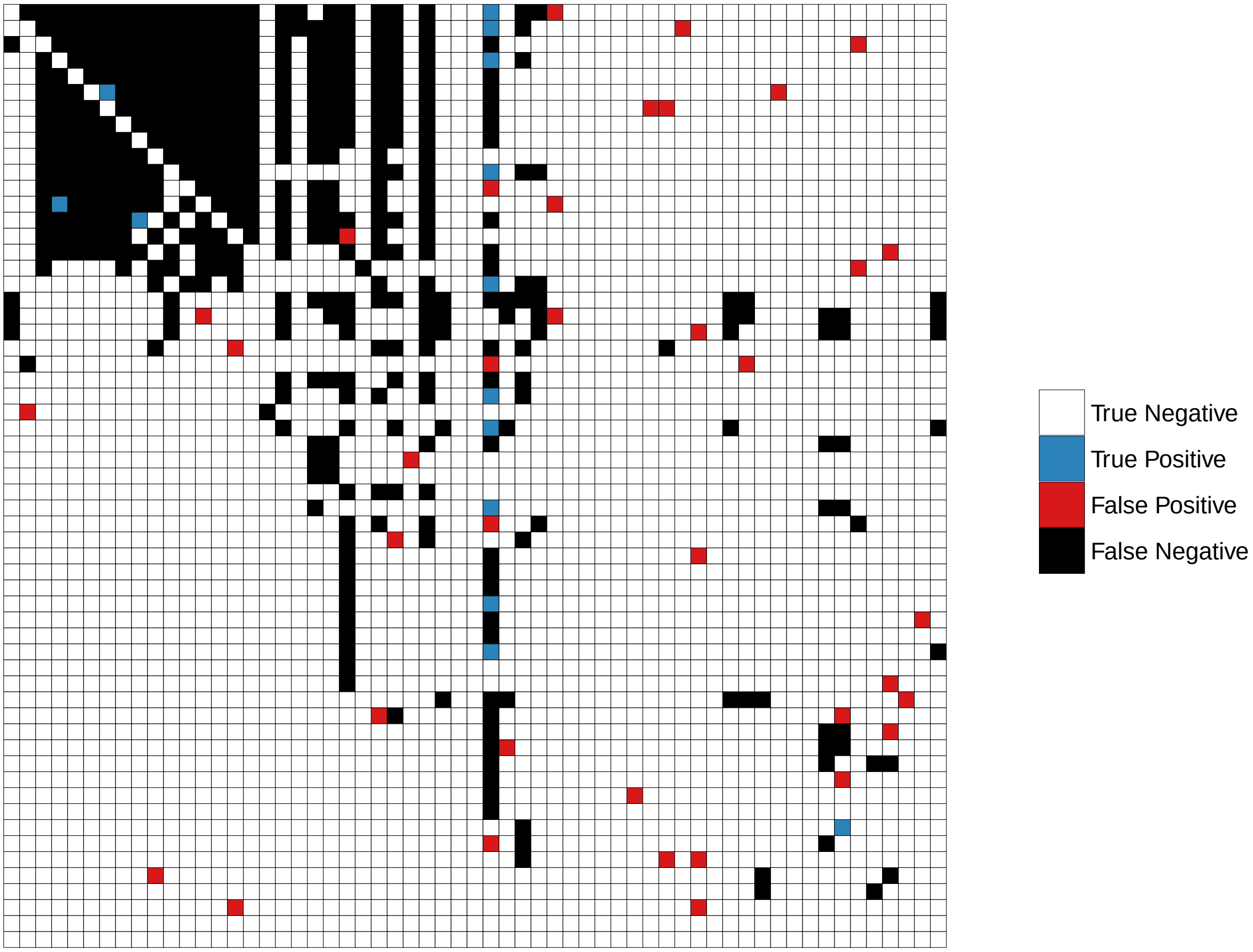

Figure S16. Structural comparison between empirical and model adjacency matrices as in Fig. 3, but for the Tatoosh nontrophic network and the DBN-4 model. Contains all species used in the DBN and Pearson models.

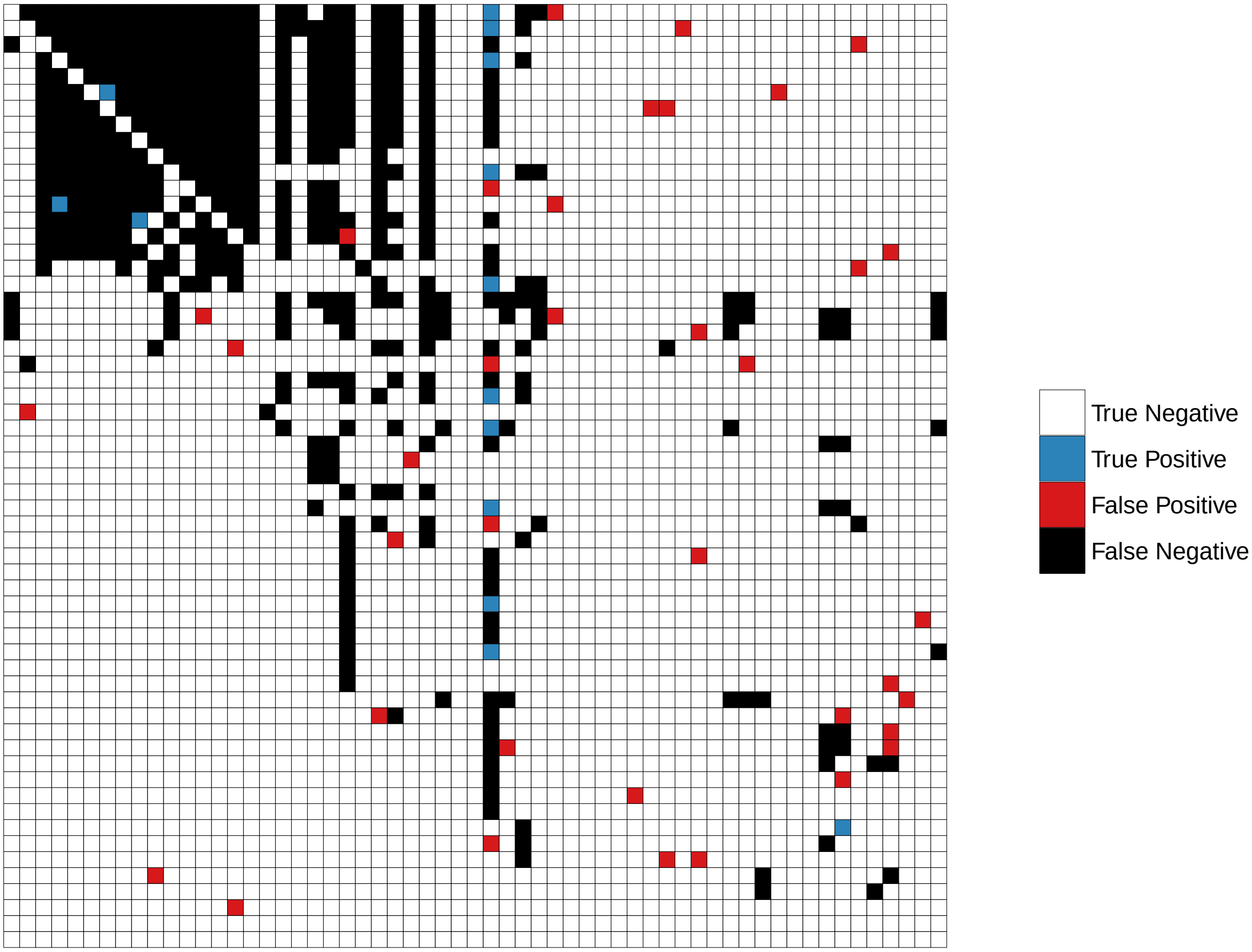

Figure S17. Structural comparison between empirical and model adjacency matrices as in Fig. 3, but for the Tatoosh nontrophic network and the DBN-5 model. Contains all species used in the DBN and Pearson models.

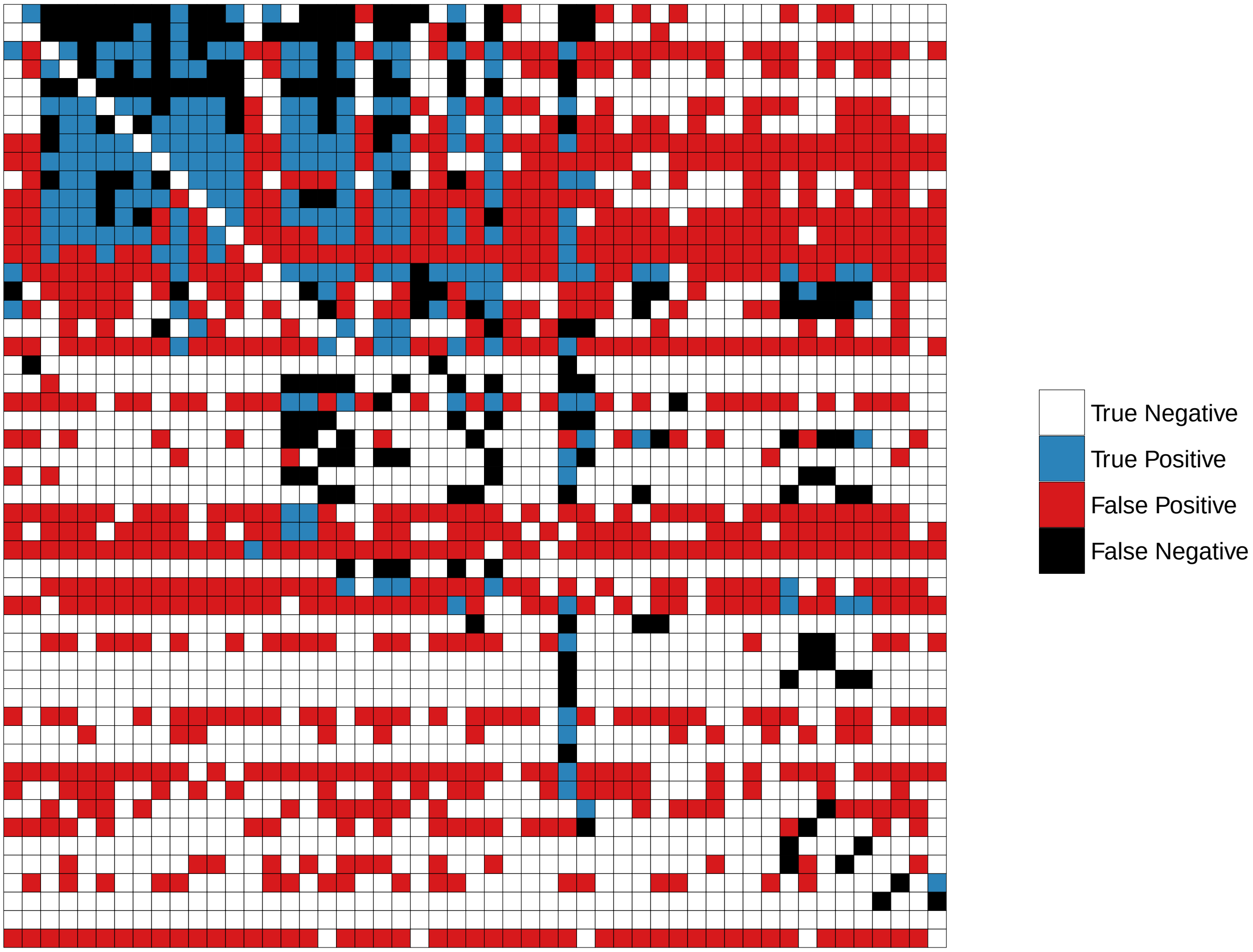

Figure S18. Structural comparison between empirical and model adjacency matrices as in Fig. 3, but for the Tatoosh nontrophic network and the Lasso-2nd model.

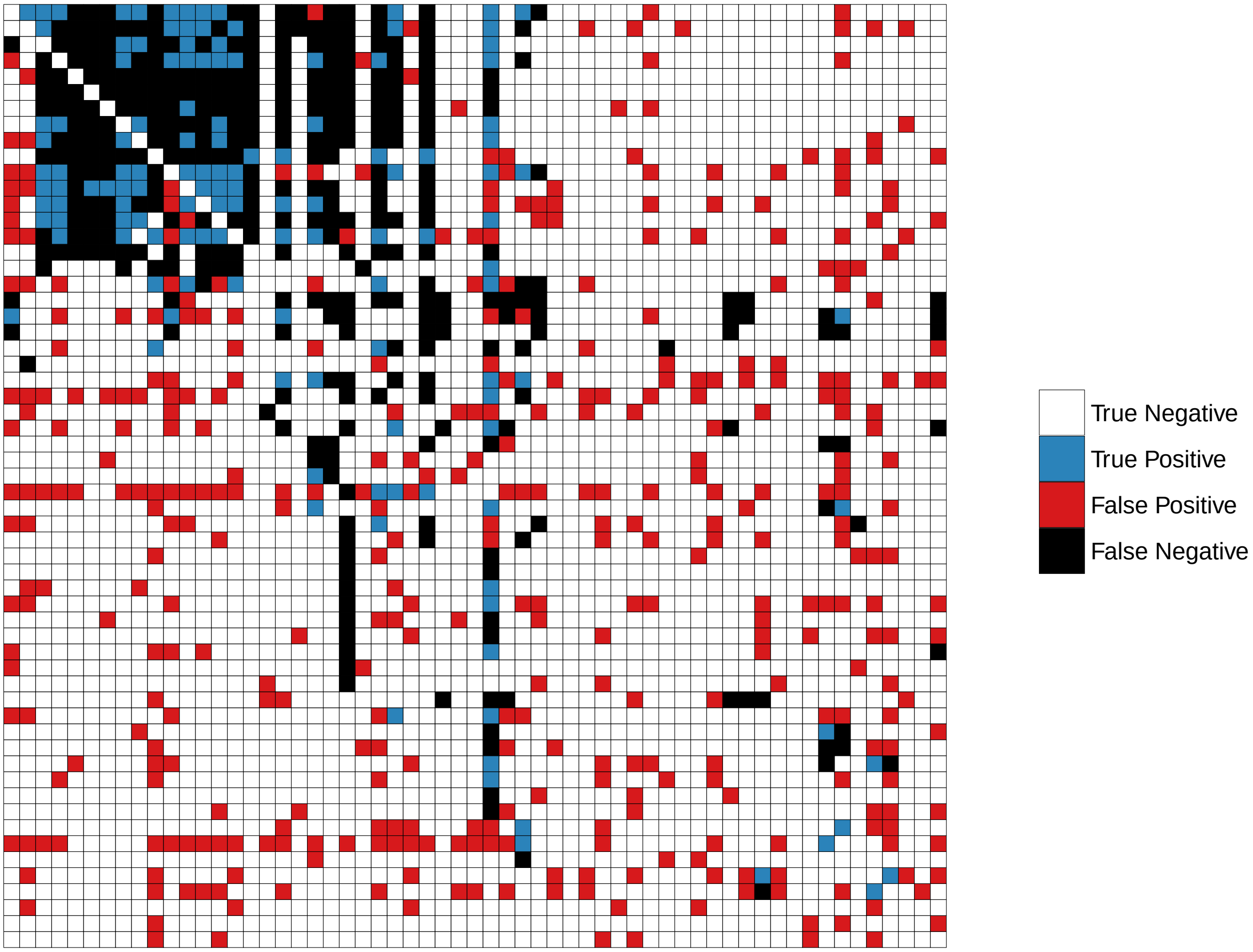

Figure S19. Structural comparison between empirical and model adjacency matrices as in Fig. 3, but for the Tatoosh nontrophic network and the Pearson model. Identical to Fig. 3f, except that it contains all species in the DBN and Pearson models, rather than excluding those which were excluded from the Lasso models.

Web	Model	Precision	Recall	p-value
Tatoosh Trophic	DBN-2	0.24	0.02	0.35
Tatoosh Trophic	DBN-3	0.15	0.01	0.78
Tatoosh Trophic	DBN-4	0.18	0.01	0.67
Tatoosh Trophic	DBN-5	0.20	0.02	0.59
Tatoosh Trophic	Lasso-1st	0.19	0.19	1.00
Tatoosh Trophic	Lasso-2nd	0.21	0.41	0.96
Tatoosh Trophic	Pearson	0.18	0.16	0.81
Tatoosh Nontrophic	DBN-2	0.31	0.03	0.02
Tatoosh Nontrophic	DBN-3	0.27	0.03	0.06
Tatoosh Nontrophic	DBN-4	0.27	0.03	0.06
Tatoosh Nontrophic	DBN-5	0.27	0.03	0.06
Tatoosh Nontrophic	Lasso-1st	0.22	0.30	0.10
Tatoosh Nontrophic	Lasso-2nd	0.20	0.53	0.12
Tatoosh Nontrophic	Pearson	0.25	0.28	0.00
France	DBN-2	0.33	0.06	0.06
France	DBN-3	0.20	0.07	0.51
France	DBN-4	0.22	0.08	0.35
France	DBN-5	0.23	0.09	0.27
France	Lasso-1st	0.24	0.57	0.02
France	Lasso-2nd	0.23	0.68	0.01
France	Pearson	0.19	0.90	0.18

Table S1. Expanded table of precision and recall with p-values. Columns represent the empirical network used, the model used to construct the inferred network, and the FDR-corrected p-values, rounded to two decimal places.

References

1. Leunda, P. M. *et al.* Feeding habits of the exotic black bullhead Ameiurus melas (Rafinesque) in the Iberian Peninsula: First evidence of direct predation on native fish species. *J. Fish Biol.* **73**, 96–114 (2008). DOI 10.1111/j.1095-8649.2008.01908.x.
2. Ogle, D. H., Selgeby, J. H., Savino, J., Newman, R. M. & Henry, M. G. Predation on Ruffe by Native Fishes of the St. Louis River Estuary, Lake Superior, 1989-1991. *North Am. J. Fish. Manag.* **16**, 115–123 (1996). DOI 10.1577/1548-8675(1996)016¡0115:PORBNF¿2.3.CO;2.
3. Radke, R. J. & Eckmann, R. Piscivorous eels in Lake Constance: can they influence year class strength of perch? *Annales Zool. Fennici* **33**, 489–494 (1996).
4. Lammens, E. H. R. R. & Visser, J. T. Variability of mouth width in European eel, Anguilla anguilla, in relation to varying feeding conditions in three Dutch lakes. *Environ. Biol. Fishes* **26**, 63–75 (1989). DOI 10.1007/BF00002476.
5. Barak, N. A.-E. & Mason, C. F. Population density, growth and diet of eels, Anguilla anguilla L., in two rivers in eastern England. *Aquac. Fish. Manag.* **23**, 59–70 (1992). DOI 10.1111/j.1365-2109.1992.tb00596.x.
6. Hartley, P. Food and Feeding Relationships in a Community of Fresh-Water Fishes. *J. Animal Ecol.* **17**, 1–14 (1948).
7. Hartley, P. The food of coarse fish, being the Interim Report on the Coarse Fish Investigation. *Sci. Publ. Freshw. Biol. Assoc. Br. Emp.* 3–33 (1940).
8. Rasmussen, G. & Therkildsen, B. Food, growth, and production of Anguilla anguilla L. in a small Danish stream. *Rapports et Proces-verbaux des Reunions Conseil Int. pour L'Exploration de la Mar* **174**, 32–40 (1979).
9. Kålås, S. The ecology of ruffe, Gymnocephalus cernuus (Pisces: percidae) Introduced to Mildevatn, western Norway. *Environ. Biol. Fishes* **42**, 219–232 (1995). DOI 10.1007/bf00004915.
10. Ogle, D. H. A Synopsis of the Biology and Life History of Ruffe. *J. Gt. Lakes Res.* **24**, 170–185 (1998). DOI 10.1016/S0380-1330(98)70811-1.
11. Dörner, H. & Benndorf, J. Piscivory by large eels on young-of-the-year fishes: Its potential as a biomanipulation tool. *J. Fish Biol.* **62**, 491–494 (2003). DOI 10.1046/j.1095-8649.2003.00035.x.
12. Frost, W. E. Observations on the Food of Eels (Anguilla anguilla) from the Windermere Catchment Area. *J. Animal Ecol.* **15**, 43–53 (1946).
13. Thomas, A. J. D. The Food and Growth of Brown Trout (Salmo trutta L .) and its Feeding Relationships with the Salmon Parr (Salmo salar L .) and the Eel (Anguilla anguilla (L .)) in the River Teify , West Wales. *J. Animal Ecol.* **31**, 175–205 (1962).
14. Larsson, P.-O. Predation on migrating smolt as a regulating factor in Baltic salmon, Salmo salar L., populations. *J. Fish Biol.* **26**, 391–397 (1985). DOI 10.1111/j.1095-8649.1985.tb04279.x.
15. Smyly, W. The Life-history of the bullhead or miller's thumb (Cottus gobio L.). *Proc. Zool. Soc. Lond.* **128**, 431–454 (1957).
16. Gaudin, P. & Calliere, L. Experimental study of the influence of presence and predation by sculpin, Cottus gobio L., on the drift of emergent brown trout, Salmo trutta L. *Arch. fur Hydrobiol.* **147**, 257–271 (2000).
17. Vøllestad, L. A., Skurdal, J. & Qvenild, T. Habitat use, growth and feeding of pike (Esox lucius L.) in four Norwegian lakes. *Arch. fur Hydrobiol.* **108**, 107–117 (1986).
18. Hartley, P. The Natural History of some British Freshwater Fishes. *Proc. Zool. Soc. Lond.* **117**, 129–206 (1947).
19. Mann, R. H. K. The Annual Food Consumption and Prey Preferences of Pike (Esox lucius) in the River Frome, Dorset. *J. Animal Ecol.* **51**, 81–95 (1982). DOI 10.2307/4312.
20. Kangur, A. & Kangur, P. Diet composition and size-related changes in the feeding of pikeperch, Stizostedion lucioperca (Percidae) and pike, Esox lucius (Esocidae) in the Lake Peipsi (Estonia). *Italian J. Zool.* **65**, 255–259 (1998). DOI 10.1080/11250009809386828.
21. Mann, R. H. K. Observations on the age, growth, reproduction and food of the pike Esox lucius (L.) in the two rivers in southern England. *J. Fish Biol.* **8**, 179–197 (1976). DOI 10.1111/j.1095-8649.1976.tb03950.x.
22. Seaburg, K. G. & Moyle, J. B. Feeding Habits, Digestive Rates, and Growth of Some Minnesota Warmwater Fishes. *Transactions Am. Fish. Soc.* **93**, 269–285 (1964). DOI 10.1577/1548-8659(1964)93.
23. Frost, W. E. The Food of Pike , Esox lucius L ., in Windermere. *J. Animal Ecol.* **23**, 339–360 (1954).

24. Adams, C. E. Shift in pike Esox lucius predation pressure following the introduction of a new fish species to Loch Lomond. *J. Fish Biol.* **38**, 663–667 (1991). DOI 10.1111/j.1095-8649.1991.tb03155.x.

25. Kekäläinen, J., Niva, T. & Huuskonen, H. Pike predation on hatchery-reared Atlantic salmon smolts in a northern Baltic river. *Ecol. Freshw. Fish* **17**, 100–109 (2008). DOI 10.1111/j.1600-0633.2007.00263.x.

26. Mauck, W. & Coble, D. The Vulnerability of Some Fishes to Northern Pike (Esox lucius) Predation. *J. Fish. Res. Board Can.* **28**, 957–969 (1971).

27. Elvira, B., Nicola, G. & Almodovar, A. Pike and red swamp crayfish: a new case on predator prey relationship between aliens in central Spain. *J. Fish Biol.* **48**, 437–446 (1996).

28. Dominguez, J. & Pena, J. C. SPATIO-TEMPORAL VARIATION IN THE DIET OF NORTHERN PIKE (Esox lucius) IN A COLONISED AREA (ESLA BASIN , NW SPAIN). *Limnetica* **19**, 1–20 (2000).

29. Allen, K. A Note on the Food of Pike (Esox lucius) in Windermere. *J. Animal Ecol.* **8**, 72–75 (1939). DOI 10.2753/JEI0021-3624430207.

30. Alp, A. *et al.* Diet composition and prey selection of the pike, Esox lucius, in Çivril Lake, Turkey. *J. Appl. Ichthyol.* **24**, 670–677 (2008). DOI 10.1111/j.1439-0426.2008.01119.x.

31. de Sostoa, A. & Lobon-Cervia, J. Observations on feeding relationships between fish predators and fish assemblages in a Mediterranean stream. *Regul. Rivers: Res. & Manag.* **4**, 157–163 (1989). DOI 10.1002/rrr.3450040208.

32. Eklöv, P. & Hamrin, S. Predatory Efficiency and Prey Selection: Interactions between Pike Esox lucius , Perch Perca fluviatilis and Rudd Scardinus erythrophthalmus. *Oikos* **56**, 149–156 (1989).

33. Mathers, R. A. & Johansen, P. H. The effects of feeding ecology on mercury accumulation in walleye (Stizostedion Vitreum) and pike (Esox lucius) in Lake Simcoe. *Can. J. Zool.* **63**, 2006–2012 (1985). DOI 10.1139/z85-295.

34. Lorenzoni, M. *et al.* Diets of Micropterus Salmoides Lac. and Esox Lucius L. in Lake Trasimeno (Umbria, Italy) and Their Diet Overlap. *Bull. Français de la Pêche et de la Piscic.* **365/366**, 537–547 (2002). DOI 10.1051/kmae:2002050.

35. Diana, J. S. The feeding pattern and daily ration of a top carnivore, the northern pike (Esox lucius). *Can. J. Zool.* **57**, 2121–2127 (1979). DOI 10.1139/z79-279.

36. Kahilainen, K. & Lehtonen, H. Piscivory and prey selection of four predator species in a whitefish dominated subarctic lake. *J. Fish Biol.* **63**, 659–672 (2003). DOI 10.1046/j.1095-8649.2003.00179.x.

37. Lawler, G. The Food of the Pike Esox lucius, in Heming Lake, Manitoba. *J. Fish. Res. Board Can.* **22**, 1357–1377 (1965).

38. Jepsen, N., Pedersen, S. & Thorstad, E. Behavioural interactions between prey (trout smolts) and predators (pike and pikeperch) in an impounded river. *Regul. Rivers: Res. & Manag.* **16**, 189–198 (2000). DOI 10.1002/(SICI)1099-1646(200003/04)16:2¡189::AID-RRR570¿3.0.CO;2-N.

39. Hunt, R. Food of Northern Pike in a Wisconsin Trout Stream. *Transactions Am. Fish. Soc.* **94**, 95–97 (1965). DOI 10.1577/1548-8659(1965)94.

40. Hynes, H. The Food of Fresh-Water Sticklebacks (Gasterosteus aculeatus and Pygosteus pungitius), with a Review of Methods Used in Studies of the Food of Fishes. *J. Animal Ecol.* **19**, 36–58 (1950).

41. Gallagher, C. P. & Dick, T. A. Winter feeding ecology and the importance of cannibalism in juvenile and adult burbot (Lota lota) from the Mackenzie Delta, Canada. *Hydrobiol.* **757**, 73–88 (2015). DOI 10.1007/s10750-015-2227-z.

42. Beeton, A. Food Habits of the Burbot (Lota lota lacustris) in the White River, a Michigan Trout Stream. *Copeia* **1**, 58–60 (1956).

43. Clemens, H. The Food of the Burbot Lota Lota Maculosa (LeSueur) in Lake Erie. *Transactions Am. Fish. Soc.* **80**, 56–66 (1951). DOI 10.1577/1548-8659(1950)80.

44. Deelder, C. A contribution to knowledge of the stunted growth of perch (Perca fluviatilis L.) in Holland. *Hydrobiol.* **3**, 357–378 (1951).

45. Pavlović, M., Paunović, M. & Simić, V. Feeding of Eurasian perch (Perca fluviatilis L.) in three reservoirs in Serbia. *Water Res. Manag.* **3**, 41–46 (2013).

46. Allen, K. The Food and Migration of the Perch (Perca fluviatilis) in Windermere. *J. Animal Ecol.* **4**, 264–273 (1935).

47. Dörner, H. *et al.* The feeding behaviour of large perch Perca fluviatilis (L.) in relation to food availability: A comparative study. *Hydrobiol.* **506-509**, 427–434 (2003). DOI 10.1023/B:HYDR.0000008608.22869.99.

48. Jamet, J.-L. Feeding activity of roach (Rutilus rutilus (L.)), perch (Perca fluviatilis, L.) and ruffe (Gymnocephalus cernuus (L.)) in eutrophic lake Aydat (France). *Aquatic Sci.* **56**, 376–387 (1994). DOI 10.1002/iroh.19940790216.

49. Estlander, S. *et al.* Diet shifts and food selection of perch Perca fluviatilis and roach Rutilus rutilus in humic lakes of varying water colour. *J. Fish Biol.* **77**, 241–256 (2010). DOI 10.1111/j.1095-8649.2010.02682.x.

50. Persson, A. & Hansson, L.-A. Diet shift in fish following competitive release. *Can. J. Fish. Aquatic Sci.* **56**, 70–78 (1999). DOI 10.1139/f98-141.

51. Musil, J. & Adámek, Z. Piscivorous fishes diet dominated by the Asian cyprinid invader, topmouth gudgeon (Pseudorasbora parva). *Biol.* **62**, 488–490 (2007). DOI 10.2478/s11756-007-0093-5.

52. Dörner, H. & Wagner, A. Size-dependent predator-prey relationships between perch and their fish prey. *J. Fish Biol.* **62**, 1021–1032 (2003). DOI 10.1046/j.1095-8649.2003.00092.x.

53. Pekcan-Hekim, Z. & Lappalainen, J. Effects of clay turbidity and density of pikeperch (Sander lucioperca) larvae on predation by perch (Perca fluviatilis). *Naturwissenschaften* **93**, 356–359 (2006). DOI 10.1007/s00114-006-0114-1.

54. Persson, L. Effects of Reduced Interspecific Competition on Resource Utilization in Perch (Perca Fluviatilis). *Ecol.* **67**, 355–364 (1986).

55. Karlsson, L., Ikonen, E., Mitans, A. & Hansson, S. The Diet of Salmon (Salmo salar) in the Baltic Sea and Connections with the M74 Syndrome. *Ambio* **28**, 37–42 (1999).

56. Jacobsen, J. A. & Hansen, L. P. Feeding habits of wild and escaped farmed Atlantic salmon, Salmo salar L., in the Northeast Atlantic. *ICES J. Mar. Sci.* **58**, 916–933 (2001). DOI 10.1006/jmsc.2001.1084.

57. Frost, W. E. River Liffey survey - II. The food consumed by the brown trout (Salmo trutta Linn.) in acid and alkaline waters. *Proc. Royal Ir. Acad. Sect. B: Biol. Geol. Chem. Sci.* **45**, 139–206 (1938). DOI 10.1017/CBO9781107415324.004. arXiv:1011.1669v3.

58. McCormack, J. C. The Food of Young Trout (Salmo trutta) in Two Different Becks. *J. Animal Ecol.* **31**, 305–316 (1962).

59. Ball, J. On the food of the brown trout of Llyn Tegid. *Proc. Zool. Soc. Lond.* **137**, 599–622 (1961).

60. Langeland, A., L'Abée-Lund, J. H., Jonsson, B. & Jonsson, N. Resource Partitioning and Niche Shift in Arctic Charr Salvelinus alpinus and Brown Trout Salmo trutta. *J. Animal Ecol.* **60**, 895–912 (1991).

61. L'Abée-Lund, J. H., Langeland, A. & Saegrov, H. Piscivory by brown trout <i>Salmo trutta</i> L. and Arctic charr <i>Salvelinus alpinus</i> (L.) in Norwegian lakes. *J. Fish Biol.* **41**, 91–101 (1992). DOI 10.1111/j.1095-8649.1992.tb03172.x.

62. Jensen, H., Kiljunen, M. & Amundsen, P.-A. Dietary ontogeny and niche shift to piscivory in lacustrine brown trout Salmo trutta revealed by stomach content and stable isotope analyses. *J. Fish Biol.* **80**, 2448–2462 (2012). DOI 10.1111/j.1095-8649.2012.03294.x.

63. Pentelow, F. The Food of the Brown Trout (Salmo trutta L.). *J. Animal Ecol.* **1**, 101–107 (1932).

64. Pedley, R. B. & Jones, J. W. The comparative feeding behaviour of brown trout, Salmo trutta L. and Atlantic salmon, Salmo salar L. in Llyn Dwythwch, Wales. *J. Fish Biol.* **12**, 239–256 (1978). DOI 10.1111/j.1095-8649.1978.tb04170.x.

65. Idyll, C. Food of Rainbow, Cutthroat and Brown Trout in the Cowichan River System, B.C. *J. Fish. Res. Board Can.* **5c**, 448–458 (1942).

66. Hyvärinen, P. & Huusko, A. Diet of brown trout in relation to variation in abundance and size of pelagic fish prey. *J. Fish Biol.* **68**, 87–98 (2006). DOI 10.1111/j.0022-1112.2006.00879.x.

67. Niva, T. Relations between diet, growth, visceral lipid content and yield of the stocked brown trout in three small lakes in northern Finland. *Annales Zool. Fennici* **36**, 103–120 (1999).

68. Lien, L. Biology of the Minnow Phoxinus phoxinus and Its Interactions with Brown Trout Salmo in Øvre Heimdalsvatn , Norway. *Holarctic Ecol.* **4**, 191–200 (1981).

69. Museth, J., Borgstrøm, R., Hame, T. & Holen, L. Å. Predation by brown trout: A major mortality factor for sexually mature European minnows. *J. Fish Biol.* **62**, 692–705 (2003). DOI 10.1046/j.1095-8649.2003.00059.x.

70. L'Abée-Lund, J. H., Aass, P. & Sægrov, H. Long-term variation in piscivory in a brown trout population : effect of changes in available prey organisms. *Ecol. Freshw. Fish* **11**, 260–269 (2002). DOI 10.1034/j.1600-0633.2002.00020.x.

71. Hvidsten, N. A. & Møkkelgjerd, P. I. Predation on salmon smolts, Salmo salar L., in the estuary of the River Surna, Norway. *J. Fish Biol.* **30**, 273–280 (1987). DOI 10.1111/j.1095-8649.1987.tb05752.x.

72. Grey, J., Thackeray, S. J., Jones, R. I. & Shine, A. Ferox trout (Salmo trutta) as 'Russian dolls': Complementary gut content and stable isotope analyses of the Loch Ness foodweb. *Freshw. Biol.* **47**, 1235–1243 (2002). DOI 10.1046/j.1365-2427.2002.00838.x.

73. Specziár, A. First year ontogenetic diet patterns in two coexisting Sander species, S. lucioperca and S. volgensis in Lake Balaton. *Hydrobiol.* **549**, 115–130 (2005). DOI 10.1007/s10750-005-5766-x.

74. Kangur, P., Kangur, A. & Kangur, K. Dietary importance of various prey fishes for pikeperch Sander lucioperca (L.) in large shallow lake Võrtsjärv (Estonia). *Proc. Estonian Acad. Sci. Biol. Ecol.* **56**, 154–167 (2007).

75. Specziár, A. Size-dependent prey selection in piscivorous pikeperch Sander lucioperca and Volga pikeperch Sander volgensis shaped by bimodal prey size distribution. *J. Fish Biol.* **79**, 1895–1917 (2011). DOI 10.1111/j.1095-8649.2011.03127.x.

76. Turesson, H., Persson, A. & Brönmark, C. Prey size selection in piscivorous pikeperch (Stizostedion lucioperca) includes active prey choice. *Ecol. Freshw. Fish* **11**, 223–233 (2002). DOI 10.1034/j.1600-0633.2002.00019.x.

77. Popova, O. & Sytina, L. Food and Feeding Relations of Eurasian Perch (Perca fluviatilis) and Pikeperch (Stizostedion lucioperca) in Various Waters of the USSR. *J. Fish. Res. Board Can.* **34**, 1559–1570 (1977).

78. Campbell, R. N. B. Food of an introduced population of pikeperch, Stizostedion lucioperca L., in lake Egirdir, Turkey. *Aquac. Fish. Manag.* **23**, 71–85 (1992). DOI 10.1111/j.1365-2109.1992.tb00597.x.

79. van Densen, W. Piscivory and the development of bimodality in the size distribution of O+ pikeperch (Stizostedion lucioperca L.). *J. Appl. Ichthyol.* **3**, 119–131 (1985).

80. Smith, P. A., Leah, R. T. & Eaton, J. W. Removal of pikeperch (Stizostedion lucioperca) from a British Canal as a management technique to reduce impact on prey fish populations. *Annales Zool. Fennici* **33**, 537–545 (1996).

81. Bergman, E. Changes in Abundance of Two Percids, Perca fluviatilis and Gymnocephalus ceruus, along a Productivity Gradient: Relations Feeding Strategies and Competitive Abilities. *Can. J. Fish. Aquatic Sci.* **48**, 536–545 (1991).

82. Ginter, K., Kangur, K., Kangur, A., Kangur, P. & Haldna, M. Diet patterns and ontogenetic diet shift of Pikeperch, Sander lucioperca (L.) fry in lakes Peipsi and Võrtsjärv (Estonia). *Hydrobiol.* **660**, 79–91 (2011). DOI 10.1007/s10750-010-0393-6.

83. Dörner, H., Hülsmann, S., Hölker, F., Skov, C. & Wagner, A. Size-dependent predator-prey relationships between pikeperch and their prey fish. *Ecol. Freshw. Fish* **16**, 307–314 (2007). DOI 10.1111/j.1600-0633.2006.00223.x.

84. Keskinen, T. & Marjomäki, T. J. Diet and prey size spectrum of pikeperch in lakes in central Finland. *J. Fish Biol.* **65**, 1147–1153 (2004). DOI 10.1111/j.1095-8649.2004.00500.x.

85. Hansson, S., Arrhenius, F. & Nellbring, S. Diet and growth of pikeperch (Stizostedion lucioperca L.) in a Baltic Sea area. *Fish. Res.* **31**, 163–167 (1997). DOI 10.1016/S0165-7836(97)00022-2.

86. Nastova-Gjorgjioska, R., Kostov, V. & Georgiev, S. Nutrition of chub Leuciscus cephalus (Linnaeaus, 1758) from the river Babuna. *Croat. J. Fish.* **55**, 53–65 (1997).

87. Ünver, B. & Erk'akan, F. Diet composition of chub, Squalius cephalus (Teleostei: Cyprinidae), in Lake Tödürge, Sivas, Turkey. *J. Appl. Ichthyol.* **27**, 1350–1355 (2011). DOI 10.1111/j.1439-0426.2011.01766.x.
